# 3D Covalent Organic Network Membranes With Regionally Ordered Nanochannels for Efficient Molecular Sieving

**DOI:** 10.1002/advs.202510911

**Published:** 2025-08-04

**Authors:** Lei Ge, Gaojing Du, Hengjie Song, Jiaqi Li, Jingwei Hou, Yatao Zhang, Junyong Zhu

**Affiliations:** ^1^ School of Chemical Engineering Zhengzhou University Zhengzhou 450001 PR China; ^2^ School of Chemical Engineering The University of Queensland St Lucia QLD 4072 Australia

**Keywords:** 3D interconnected nanochannels, accurate molecular sieving, covalent organic network (CON) membrane, organic solvent nanofiltration

## Abstract

Polymeric composite membranes hold significant promise for molecular sieving in organic solvents, yet struggle with the precise fractionation of small molecules, a critical need in the chemical and pharmaceutical industries. Here an innovative covalent organic network (CON) membrane featuring 3D interpenetrated ultra‐microporous channels for effective fractionation of small‐molecule solutes is presented. Rational utilization of unique tetrahedral amine (TAM) as aqueous‐phase building blocks to crosslink with acyl chlorides yields CON polyamide thin films with finely tuned ordered porosity. Importantly, three‐node acyl chloride (TMC) with high reactivity facilitates sharpening the membrane pore size distribution due to the partially ordered and elevated crosslinked structure. Molecular dynamics simulations reveal that these TAM‐TMC membranes possess a high fractional free volume, contributing to superior permeability‐selectivity performance. Further post‐activation with polar solvents effectively modulates the inherent pore polarity and width, resulting in an exceptional methanol permeability of 7.5 L m^−2^ h^−1^ bar^−1^ and a molecular weight cut‐off of 248 Da. This ultrafine 3D hierarchical pore architecture enables excellent fractionation of dye mixtures with similar molecular weights and efficient antibiotic concentration. This work rationalizes the design of chemically stable covalent organic network membranes with 3D partially ordered sub‐microporosity, extending their application to precision molecular separations.

## Graphical abstract

Precise control of acyl chloride monomers to modulate the free volume of 3D covalent organic network membranes. Acid‐induced of confined reactions and promotes the controlled arrangement of polyamide (PA) chains into semi‐crystalline PA structures. The solvent activation technique is used to improve the pore environment and optimize the thickness of the selective layer, and the activated PA membrane has excellent solvent permeability and high‐precision fractionation performance.

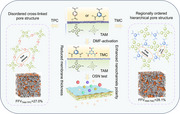

## 1 Introduction

As global demand for organic solvents surges across pharmaceutical, chemical, energy, and environmental industries, efficient solvent separation becomes increasingly critical.^[^ ^1^ ^]^ Organic solvent nanofiltration (OSN) technology offers a transformative, energy‐efficient, and low‐carbon footprint solution for molecular separations in organic systems.^[^ ^2^ ^]^ As an effective alternative to conventional distillation and extraction, OSN can reduce energy consumption by over 90%.^[^ ^3^ ^]^ Presently, the design principles for high‐performance OSN membranes prioritize: i) robust physicochemical stability; ii) narrow pore size distribution and a small molecular weight cut‐off (MWCO) below 300 g mol^−1^; iii) an ultrathin and highly microporous selective layer.^[^ ^4^ ^]^

The application of OSN technology is largely constrained by a permeability‐selectivity trade‐off in conventional linear polymeric membranes, stemming from excessive cross‐linking, limited microporosity, and weak pore interconnectivity.^[^ ^5^ ^]^ The advent of microporous organic polymers (MOPs) offers a powerful strategy to overcome these limitations. The molecular‐level design of robust void‐forming elements endows MOPs with permanent and well‐defined micropore structures, intrinsically restricting chain mobility and stacking. This characteristic, alongside their tunable pore architecture and chemistry, positions them as ideal candidates for fabricating MOP‐based membranes with precisely controlled pore sizes for task‐specific separations.^[^ ^6^ ^]^ Moreover, the planar building blocks of 2D‐MOP (e.g., covalent organic framework [COF]) membrane can assemble into anisotropic 3D frameworks via π–π interactions, yielding interlayer sub‐nanochannels that facilitate the precise differentiation of molecules even with similar dimensions.^[^ ^7^ ^]^ Nevertheless, the directional synthesis of 2D‐layered membranes is significantly hampered by challenges in simultaneously controlling covalent in‐plane growth and supramolecular out‐of‐plane assembly.^[^ ^8^ ^]^ In contrast, fully covalent 3D architectures offer a compelling solution, enabling multidimensional polymerization to create interconnected polymeric nanochannels.^[^ ^9^ ^]^ Constructed from tetrahedral and linear building blocks, these 3D covalent networks intrinsically form open channels spanning all spatial dimensions, thereby facilitating stable solvent transport. Meanwhile, the resultant interpenetrating network establishes cage‐like micropores, which present a promising avenue for the efficient rejection of low‐molecular‐weight organic compounds. Consequently, precise molecular‐level tailoring of pore architecture and ultrafine pore aperture within 3D MOP membranes for rapid and effective precision sieving can be achieved through the coherent integration of non‐planar monomer building blocks and/or post‐synthetic modifications.^[^ ^10^ ^]^

3D MOP membranes with interconnected nanochannels are primarily categorized into amorphous polymeric and crystalline COF membranes. Amorphous polymeric thin membranes are often synthesized via interfacial crosslinking of twisted building blocks.^[^ ^11^ ^]^ For instance, Pinnau et al. reported the synthesis of fully aromatic polyamide membranes using bridged‐bicyclic triptycene tetraacyl chloride, which achieved precise sub‐microporosity and high performance in the removal of small organic microcontaminants.^[^ ^12^ ^]^ Although twisted aromatic acyl chlorides facilitate structural rigidity and microporosity, their intrinsic flexibility and reactivity inevitably induce rapid and random crosslinking. Consequently, this can result in inefficiently filled interconnecting micropores, ensuring precise small molecule separation but often at the expense of liquid permeance in 3D amorphous polymers. In contrast, 3D COF membranes, characterized by their periodic organic frameworks and multi‐directionally interconnected nanochannels, provide efficient molecular transport pathways and enhanced accessibility to active sites.^[^ ^13^ ^]^ Shi et al. reported the fabrication of 3D COF membranes from tetra(4‐formylphenyl)methane and hydrazine hydrate, yielding sub‐nanopores and interpenetrated nanochannels that simultaneously improved selectivity and permeability.^[^ ^14^ ^]^ Nonetheless, critical challenges remain in controlling the crystallinity of thin 3D‐COF films and in finely regulating their formation in the IP reaction.^[^ ^15^ ^]^ Additionally, the progress in 3D MOP membrane development currently lags behind. To address these limitations, we aim to construct partially crystallized 3D amorphous polymeric membranes. Consequently, we propose constructing partially crystallized 3D‐MOP membranes to boost solvent transport while simultaneously maintaining amorphous crosslinked regions to ensure high selectivity.

In this context, we report the synthesis of 3D partially ordered and highly microporous covalent organic network (CON) films via confined interfacial polymerization (IP) of tetrahedral tetra‐(4‐aminophenyl) methane (TAM) with acyl chloride monomers (trimesoyl chloride: TMC or terephthaloyl chloride: TPC). The tetrahedral architecture of TAM introduces steric hindrance and reactive amine groups. When combined with the altered geometry of acyl chloride groups, these features enable precise modulation of microporosity and free volume within 3D‐CON membranes. Moreover, the introduction of hydrochloric acid into the aqueous phase is capable of decelerating amide reaction kinetics, given its presence as a by‐product that affects the reaction environment.^[^ ^16^ ^]^ This controlled polymerization facilitates the creation of thin nanofilms with high pore interconnectivity and promotes crystalline region formation. Experimental and simulation results reveal that TAM‐TMC membranes exhibit a partially ordered structure, sharper pore size distribution, and higher free volume fraction than TAM‐TPC counterparts. Despite further activation with the polar solvent DMF, TAM‐TMC membranes maintained their semi‐crystallinity with negligible alteration. Crucially, the activated TAM‐TMC membranes delivered exceptional methanol permeability and precise small‐molecule sieving capabilities, culminating in their successful deployment for antibiotic concentration and purification. This work paves the way for the design of 3D‐CON membranes with partially crystalline regions for accurate and rapid molecular separation.

## 2 Results and Discussion

### 2.1 Fabrication and Characterization of 3D CON Membranes

**Figure 1:**
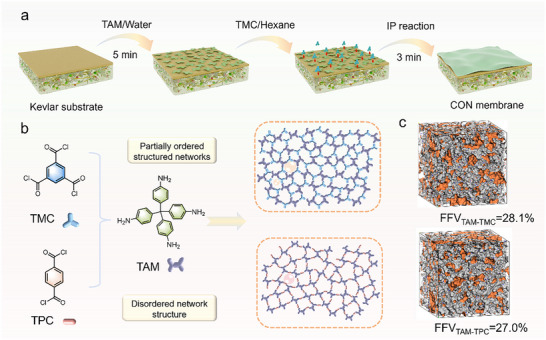
Synthesis of 3D CON membranes. a) Schematic illustration of the IP reaction to fabricate microporous CON membranes between TAM in the aqueous solution and TMC/TPC in the oil phase; b) Microstructure diagram of the TAM‐TMC/TPC nanofilms; c) 3D views of amorphous models containing TAM‐TMC and TAM‐TPC polymer. Gray color: accessible porous surface.

Molecular transport in thin‐film composite (TFC) membranes is predominantly determined by the thickness and nanoscale uniformity of the formed selective layer. These crucial features can be optimized by precisely altering IP parameters. In specific, the initial dispersion of amine monomers in the aqueous phase and their diffusion kinetics across the organic‐aqueous interface are critical in dictating both the growth and structural characteristics of the selective layer nanofilms.^[^ ^17^ ^]^ Herein, we fabricated polyamide‐based covalent organic network (CON‐PA) nanofilms using tetrahedral TAM and planar acyl chlorides (Figure **1**a; Figure S1, Supporting Information). Here, the addition of hydrochloric acid (HCl) not only enhances TAM solubility in the aqueous phase, but also enables the restrained IP to yield high‐free‐volume polyamide membranes. This restrained IP is attained via physical inhibition of protonated TAM diffusion and chemically retarded amide reaction at the presence of by‐product HCl.^[^ ^18^ ^]^ Using pulse‐gradient spin‐echo nuclear magnetic resonance (NMR), we compared the diffusion coefficients of TAM with and without HCl addition.^[^ ^19^ ^]^ The results showed that protonated TAM exhibited a lower diffusion coefficient than TAM (Figure S2, Supporting Information). The dynamic diffusion experiments further revealed the lower absorbance of protonated TAM at the water/hexane interface over the same time framework (Figure S3, Supporting Information). This signifies that a diminished number of protonated TAM diffuse to react with TMC monomers within the same time framework compared to TAM, leading to a limited polymerization degree. On the other hand, serving as a typical by‐product of amide reaction, the substantial presence of HCl can impede the progression of the forward reaction, chemically restraining the amide condensation. This physical and chemical synergistic effect eventually enables the formation of moderately crosslinked polyamide networks that feature high free volume, regionally ordered nanochannels, and superior pore interconnectivity (Figure 1b). The unique molecular architecture and sterically induced non‐planar conformation of TAM, combined with modulation of acyl chloride groups, enables precise control over the free volume and ordered structure of the CON membrane. Molecular dynamics (MD) simulations provide conclusive evidence of the highly microporous nature of the resulting CON‐PA nanofilms, exhibiting fractional free volume (FFV) values of 28.1% for TAM‐TMC and 27.0% for TAM‐TPC configurations (Figure 1c). Importantly, substituting TPC with TMC building blocks induces a substantial increase in the void volume of the covalent organic polymer, significantly correlating with enhanced pore interconnectivity for improved molecular transport.^[^ ^20^ ^]^

### 2.2 Design, Fabrication, Morphology, and Structure of Free‐Standing Nanofilms

**Figure 2:**
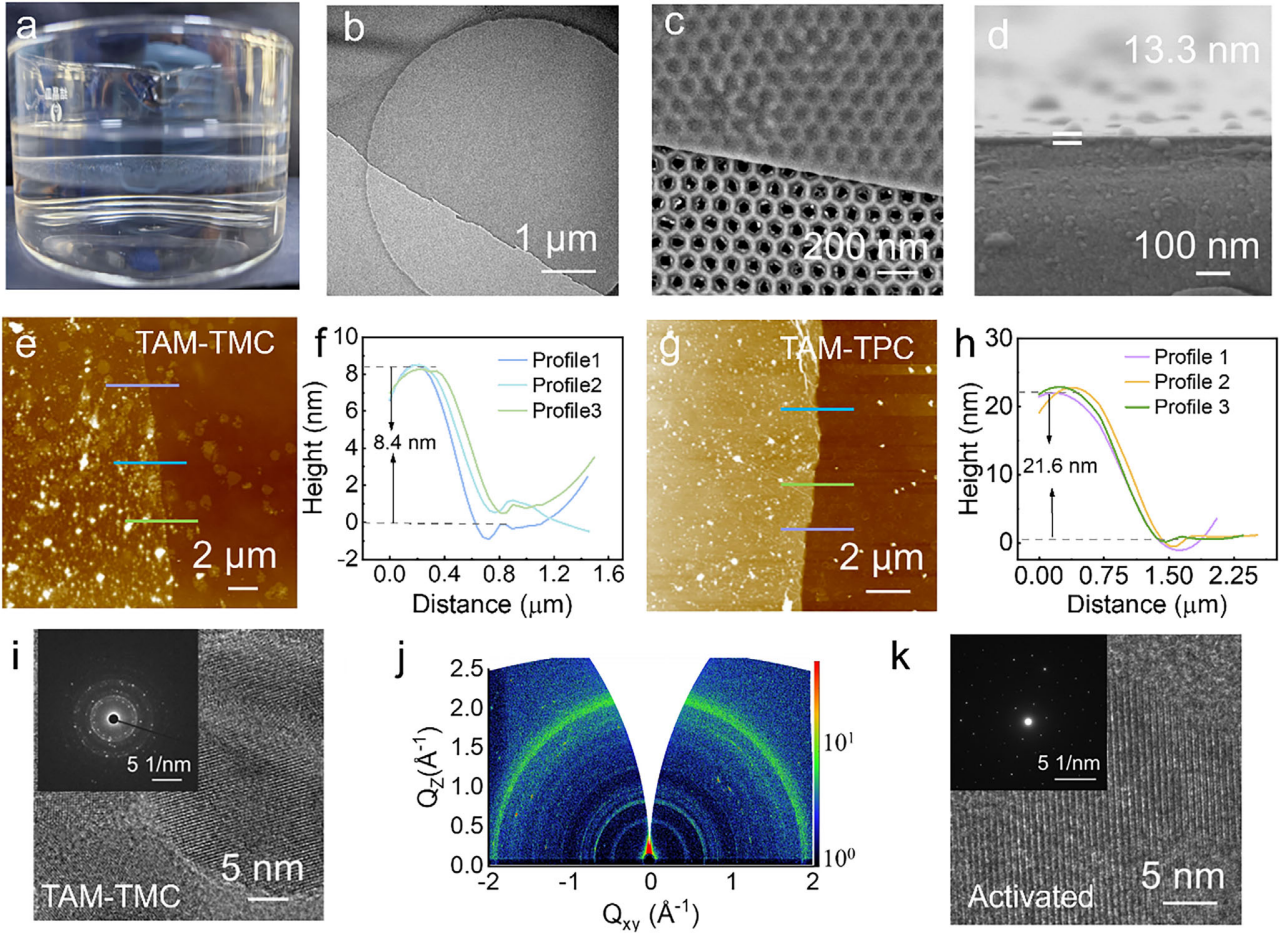
Morphology and characteristics of PA nanofilms prepared via IP. a) The image of free‐standing nanofilms formed at the water‐oil interface; b) TEM image of the surface PA nanofilm of copper mesh; c) SEM image of the transfer PA nanofilm on the surface of anodic aluminum oxide (AAO); d) SEM images of TAM‐TMC free‐standing nanofilms loaded on slides; e–h) The AFM image depicts and the height profile of the TAM‐TMC nanofilm and TAM‐TPC nanofilm on the silicon wafer; i) HR‐TEM images of TAM‐TMC nanofilms; j) GIWAXS image of the TAM‐TMC nanofilms; k) HR‐TEM images of activated TAM‐TMC nanofilms.

**Figure** **2**a illustrates the free‐standing nanofilms synthesized via IP reaction of TAM (0.2 w/v%) and TMC (0.1 w/v%) at the oil‐water interface within 3 min. The collected nanofilms were subsequently transferred onto various substrates for comprehensive characterization. High‐resolution transmission electron microscopy (HR‐TEM) and scanning electronmicroscope (SEM) images revealed the translucent, continuous, and defect‐free structure of the free‐standing TAM‐TMC nanofilms (Figure 2b,c; Figure S4, Supporting Information). Energy dispersive spectrometer mapping further demonstrated a uniform distribution of carbon, nitrogen, and oxygen across the TAM‐TMC nanofilms (Figure S5, Supporting Information). Similarly, TAM‐TPC nanofilms exhibited comparable structural integrity (Figure S6, Supporting Information). For an intuitive observation, the nanofilm was transferred onto a coverslip following by being fractured in liquid nitrogen for SEM characterization. The thickness of TAM‐TMC nanofilm in Figure 2d was ≈13.3 nm. Atomic force microscopy (AFM) analysis revealed an average film thickness of ≈8.4 nm for the TAM‐TMC nanofilm (Figure 2e,f). In contrast, TAM‐TPC nanofilms had a higher thickness of 26.7 nm via SEM analysis (Figure S7, Supporting Information), consistent with the value determined by AFM height profile measurements (Figure 2g,h). TAM‐TMC nanofilms exhibited clear and well‐defined lattice fringes in HR‐TEM and a polycrystalline selected‐area electron diffraction (SAED) pattern (inset) (Figure 2i), indicative of partially ordered nanostructures formed during IP. Conversely, TAM‐TPC films displayed an amorphous HR‐TEM structure (Figure S8, Supporting Information). Grazing incidence wide‐angle X‐ray scattering (GIWAXS) patterns displayed more pronounced Debye‐Scherrer rings, indicating preferential orientation and partial crystallinity (Figure 2j). The discontinuous diffracted spot of TAM‐TPC, consistent with its amorphous HR‐TEM image (Figure S9, Supporting Information), confirmed its disordered structure.^[^ ^21^ ^]^ This ordering is hypothesized to arise from the controlled growth and assembly of PA chain segments during the confined IP reaction.

Solvent activation technique using N'N dimethylformamide (DMF) is a facile and effective method to boost membrane permeability of polyamide membranes by precisely modulating the microenvironments of their microporous structures.^[^ ^4^, ^22^ ^]^ DMF induces relaxation and structural rearrangement of polyamide chain segments. The formed low‐molecular‐weight oligomers and unreacted segments within the selective layer can be feasibly removed after DMF activation, thereby providing additional transport nanochannels while retaining the highly crosslinked polyamide networks.^[^ ^23^ ^]^ HR‐TEM images revealed that the activated TAM‐TMC membrane maintained well‐defined lattice fringes, with the SAED pattern displaying distinct diffraction spots (Figure 2k). These results collectively confirm that TAM‐TMC membranes maintain well‐defined regionally crystalline regions.

### 2.3 Morphology and Characterization of 3D CON Membranes

**Figure 3:**
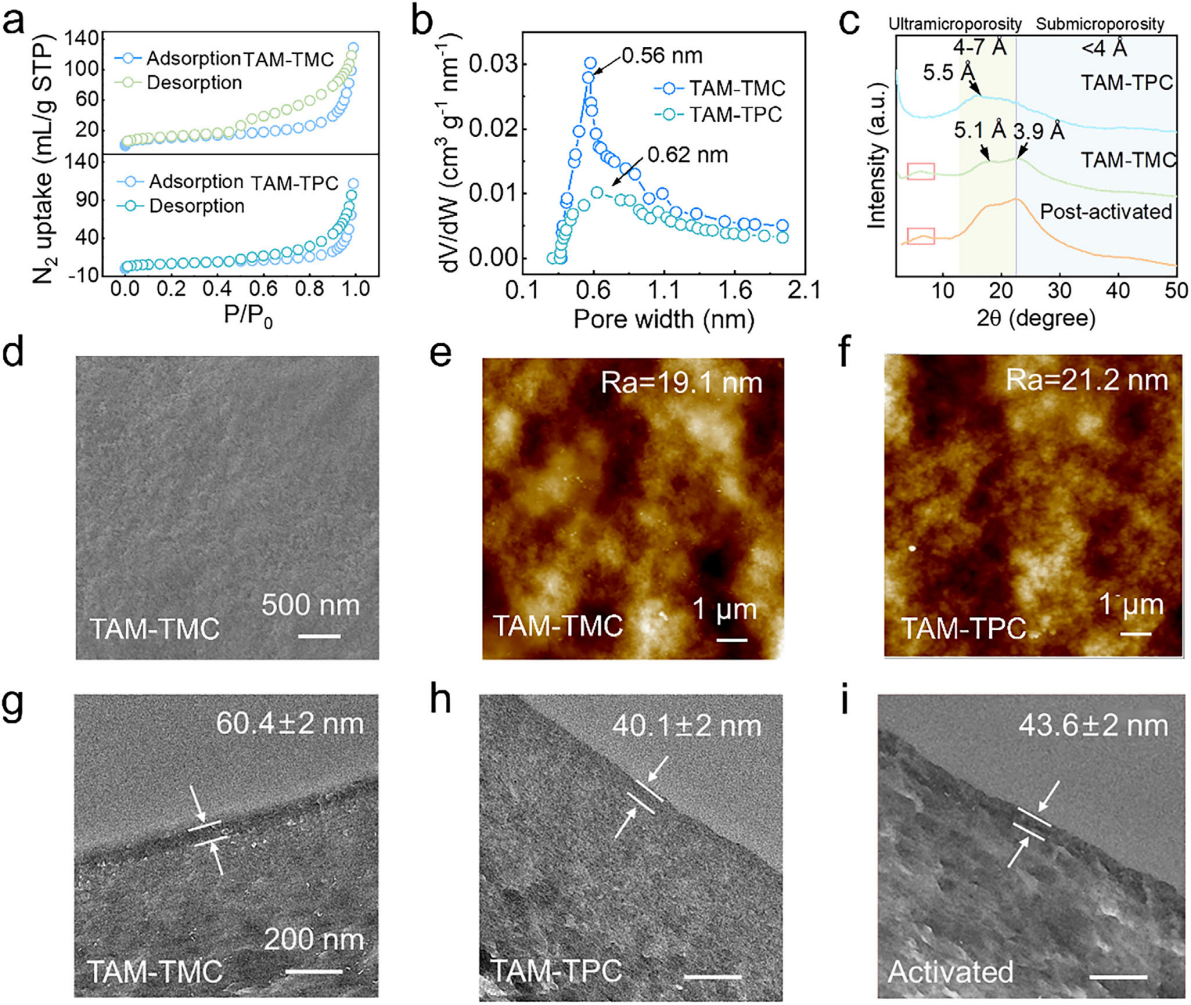
Chemical structure characterization of the 3D CON nanofilms/TFC membranes. a,b) Nitrogen adsorption−desorption isotherms of the PA film and the corresponding pore size distribution; c) XRD spectra of CON‐PA nanofilms with average d‐spacing values calculated by Bragg's law; d) Surface SEM images of TAM‐TMC composite membrane; e,f) Surface AFM images of the TAM‐TMC/TPC composite membrane; g–i) Cross‐sectional TEM image of the different PA‐TFC membrane (scale bar: 200 nm).

Nitrogen adsorption‐desorption isotherms and pore size distribution analysis revealed significant differences between the two PA nanofilms (**Figure** **3**a,b). The TAM‐TMC nanofilm exhibited a higher BET surface area of 40.2 m^2^ g^−1^, in contrast to 24.5 m^2^ g^−1^ for TAM‐TPC. Non‐local density functional theory (NLDFT) analysis further showed that the TAM‐TMC nanofilm possessed a narrower pore size distribution centered at 0.56 nm, while TAM‐TPC had a peak at 0.62 nm. Powder X‐ray diffraction (PXRD) provided further structural insights (Figure 3c). TAM‐TMC nanofilms displayed characteristic diffraction peaks at 6°, 17.2°, and 22.8°. Peak deconvolution analysis of the XRD data revealed a crystallinity of 59.8% for the TAM‐TMC nanofilms, which strongly confirmed the presence of a partially ordered crystalline structure.^[^ ^24^ ^]^ Bragg's law calculations for the 17.2° and 22.8° peaks corresponded to d‐spacings of 0.51 and 0.39 nm, suggesting a partially ordered, potentially hierarchical pore structure within TAM‐TMC that facilitates selective molecular transport. Conversely, TAM‐TPC nanofilms exhibited an amorphous nature, with a broad average chain d‐spacing of 0.55 nm and a disordered PXRD pattern. Additionally, the DMF‐treated nanofilms exhibited characteristic diffraction peaks at 6.5°, 17.8°, and 22.5°, which were nearly identical to those of the pristine TAM‐TMC nanofilms. This confirms that the partially ordered regions of the CON‐PA nanofilms remain intact after DMF treatment.

Utilizing the nanofilm formation properties at the water‐hexane interface, we prepared and characterized CON‐TFC membranes on Kevlar substrates (Figure 1a; Figure S10, Supporting Information). SEM analysis of the TAM‐TMC composite membranes revealed a continuous and smooth surface morphology (Figure 3d). This smooth film growth is attributed to the non‐coplanar structure of the TAMs, which induces spatial site resistance and decelerates its diffusion kinetics relative to conventional amine monomers (e.g., 2,5‐Diaminotoluene: MPD and piperazine: PIP), facilitating a more continuous and uniform nanofilm. In contrast, the TAM‐TPC composite membrane exhibited a denser surface with granular protrusions. This morphology suggests an initial disordered cross‐linking reaction between TAM and TPC, leading to uncontrolled monomer transport and subsequent polymer buildup. This finding aligns with the change in surface roughness of TFC membrane surface measured by AFM (Figures 3e,f). The primary distinction lies in monomer structural variability: TMC that features higher reactivity and more crosslinking sites than TPC, reacts with acid‐protonated amine monomers at an optimized rate. This results in the formation of a partially ordered polymeric structure within the crosslinked network. To further investigate this microstructural evolution, we conducted monomer concentration‐dependent SEM and AFM studies on the TFC membranes. Compared to the pristine Kevlar substrate (Figure S11, Supporting Information), increasing TAM concentration at fixed TMC concentrations (0.1 w/v%) resulted in the gradual flattening of initially raised granular nodules on the membrane surface (Figures S12 and S13, Supporting Information). This morphological change is attributed to the acid‐induced chemical confined IP reaction dominating the film‐forming process.^[^ ^25^ ^]^ Initially, PA nuclei form prominent nodular structures on the Kevlar substrate. As amine monomer concentration increases, enhanced nucleophilic addition reactions promote structural densification of the polymer network. These forming particles gradually compensate for surface defects, leading to a decrease in membrane roughness (Figure S14, Supporting Information). Similarly, as either TMC concentration or the TAM/TMC ratio increased, the initially observed anchored polymer nanoparticles on the membrane surface evolved into a continuous nanofilm. This transition was marked by a shift from protruding particle structures to a smooth surface layer (Figures S15–S17, Supporting Information). In contrast, for TPC‐regulated composite membranes, increasing TPC concentration promoted the rapid accumulation of subsequently formed polymer particles on the initial membrane surface, leading to enhanced surface roughness (Figure S18, Supporting Information). TEM images revealed the selective layer thicknesses of ≈60.4 nm for TAM‐TMC membranes and 40.1 nm for TAM‐TPC membranes (Figures 3g and 3h), consistent with cross‐sectional SEM imaging data (Figure S19, Supporting Information). The thickness difference, compared to free‐standing nanofilms, arises from the dense film formed during initial reaction at the free water‐oil interface, which acts as a barrier to limit monomer diffusion and crosslinking. In contrast, hydrogen‐bonding and electrostatic interactions between the Kevlar hydrogel substrate and tetramine facilitated the formation of initially loose‐structured nanofilms, which allowed for sustained monomer supply and subsequent film growth.^[^ ^26^ ^]^

The SEM analysis of TAM‐TMC activated with DMF for varying durations (Figure S20, Supporting Information), showed that prolonged treatment had a negligible effect on their surface morphologies, consistent with minor variations in their surface roughness after 1 h of DMF treatment (Figure S21, Supporting Information). TEM analyses of TAM‐TMC composite membranes before and after DMF treatment revealed an activated selective layer thickness of ≈43.6 nm, highly consistent with the layer thickness characterized by SEM imaging (Figure 3i; Figure S22, Supporting Information). This observed thickness reduction stems from the dissolution of discontinuous nanostructures in the pristine membrane by DMF, ultimately forming a highly porous selective layer.^[^ ^27^ ^]^

### 2.4 Physicochemical Properties of 3D CON Membranes

**Figure 4:**
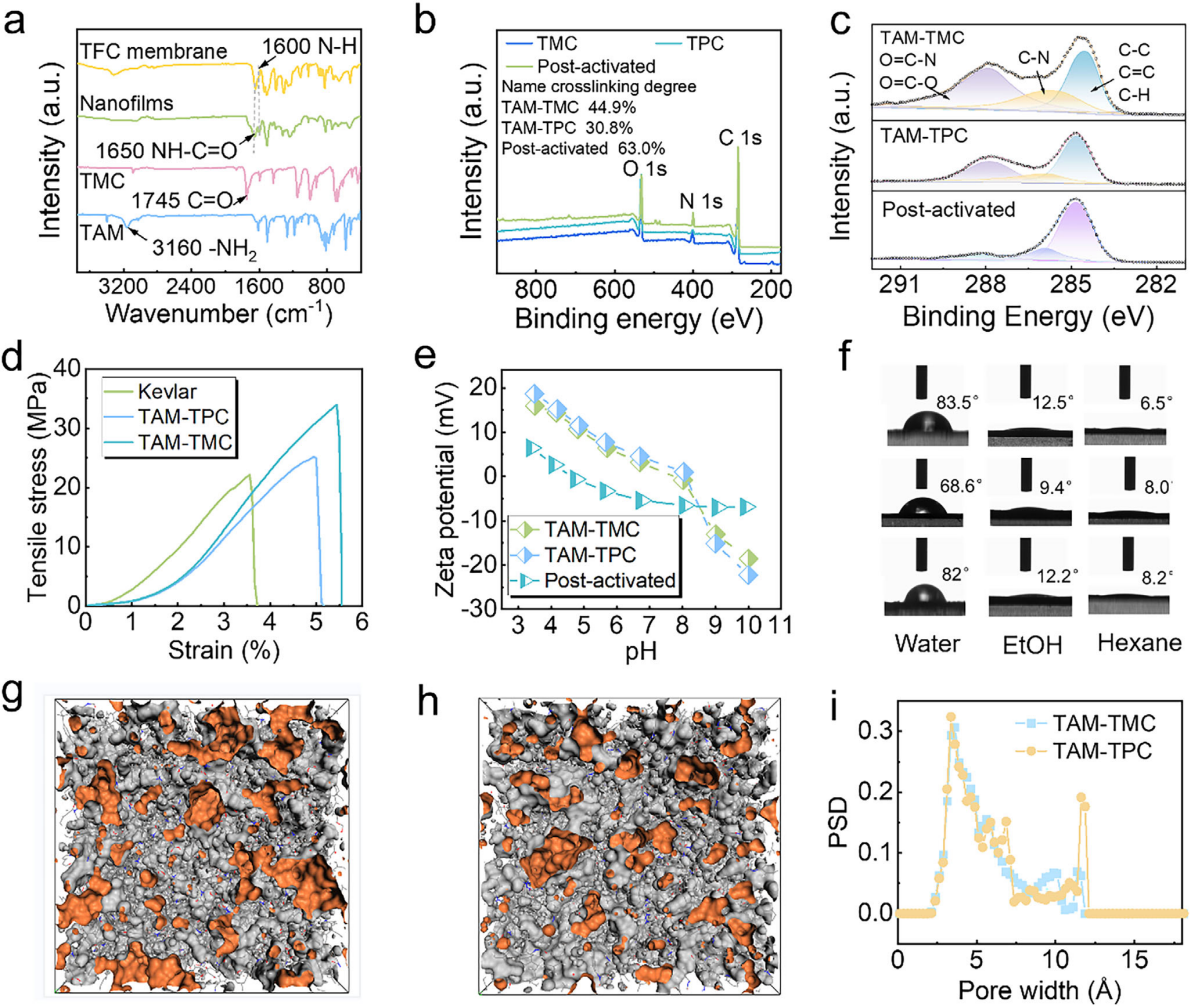
Physicochemical properties of the 3D CON membranes: a) FT‐IR spectra; b) XPS spectra and c) C1s spectra of the CON‐PA membranes; d) Mechanical properties testing. Stress‐strain curves of the Kevlar substrate and PA membrane; e) Zeta potential of the Kevlar and PA membrane surfaces at different pH values; f) The contact angle (CA) variation different solvent on the top surface of PA membrane (from top to bottom: TAM‐TMC membrane; TAM‐TPC membrane; Post‐activated TAM‐TMC membrane); g,h) Top‐down view of TAM‐TMC and TAM‐TPC polyamides; i) PSD plot of TAM‐TMC and TAM‐TPC polymer.

The surface chemical structure of CON‐PA membranes was analyzed by attenuated total reflectance‐Fourier transform infrared spectroscopy (ATR‐FTIR) (**Figure** **4**a). After IP reaction, the significant attenuation of O═C─Cl (1745 cm^−1^) and ‐NH_2_ (3160 cm^−1^) stretching bands, along with the appearance of the NH─C═O characteristic band at 1650 cm^−1^, confirmed the formation of amide bonds through IP reaction.^[^ ^28^ ^]^ A similar successful crosslinking was observed for between TAM and TPC monomers (Figure S23, Supporting Information). While both DMF‐activated and pristine TAM‐TMC membranes exhibited identical FT‐IR spectra (Figure S24, Supporting Information), the attenuated NH─C═O peak in activated membranes indicated a severe DMF‐induced swelling and partial etching of the polyamide layer.^[^ ^27^, ^29^ ^]^ X‐ray photoelectron spectroscopy (XPS) spectra (Figures 4b,c) revealed that all TFC membranes displayed characteristic O 1s (532.1 eV), N 1s (400.8 eV), and C 1s (284.8 eV) peaks. Deconvolution of the C 1s fine spectrum into N─C (286.3 eV), C═C (284.8 eV), and C═O─N (287.8 eV) components confirmed the formation of PA structures through confined IP. No significant chemical composition differences were detected among the three PA membranes (Figures S25–S28, Supporting Information). Quantitative XPS analysis revealed crosslinking degrees of 44.9%, 30.8%, and 63.0% for TAM‐TMC, TAM‐TPC, and activated TAM‐TMC membranes, respectively (Figure 3b; Table S1, Supporting Information). These results demonstrate that TAM‐TMC membranes possess a more compact and densely crosslinked PA layer than TAM‐TPC, contributing to their enhanced molecular selectivity. The increased crosslinking in DMF‐activated membranes is attributed to DMF selectively etching looser surface structures without affecting the denser regions, thus effectively increasing the crosslinking density of the remaining PA network.^[^ ^23^ ^]^

Thermogravimetric analysis (TGA) confirmed the excellent thermal stability of CON‐PA membranes under nitrogen atmosphere, exhibiting degradation temperatures exceeding 400 °C (Figure S29, Supporting Information). Mechanical stability tests using a universal testing machine revealed that the TAM‐TMC membrane possessed superior properties, with a tensile strength of 33.9 MPa, elongation at break of 5.5%, and Young's modulus of 516.6 MPa (Figure S30, Supporting Information). These values significantly surpassed those of both the TAM‐TPC membrane and the Kevlar substrate, demonstrating their exceptional pore structure rigidity, crucial for stable and precise molecular transport in organic liquids.^[^ ^30^ ^]^ Zeta potential measurements elucidated the charge characteristics of CON‐PA membranes across a wide pH range (Figure 4e). Notably, the membrane surface had a reduced value of ζ‐potential after DMF treatment, indicates stronger electronegativity of activated polyamide membranes. Furthermore, the water contact angles (WCAs) of CON‐PA layers can be feasibly tuned by varying monomer concentration (Figures S31–S33, Supporting Information). As shown in Figure 4f, the CAs of TAM‐TMC membranes for water, ethanol, and hexane were 83.5°, 12.5°, and 6.5°, respectively. This indicates a high affinity for organic solvents and suggests a preference for the transport of low‐polarity organic solvents (Figures S34 and S35 and Table S2, Supporting Information). The organophilic characteristic of the CON‐PA membrane surface is mainly attributed to the intrinsically hydrophobic aromatic backbone. In addition, DMF‐treated TAM‐TMC membranes exhibit elevated surface hydrophilicity and more negatively charged surfaces. The enhanced abundance of polar functional groups within the membrane pores strengthened affinity for polar solvents through dipole‐dipole interactions, while simultaneously increasing mass transfer resistance for nonpolar solvents. This resulted in a decreased contact angle for methanol and an increased contact angle for n‐hexane following DMF treatment (Figures S36 and S37, Supporting Information).^[^ ^31^ ^]^

### 2.5 Molecular Simulation of 3D CON Polyamides

To elucidate the pore architecture of the CON network, we performed molecular simulations to generate realistic structural models and analyze their properties (detailed information in SI). The 3D top‐view models show interconnected voids (yellow regions) within both TAM‐TMC and TAM‐TPC, based on a theoretical probe radius of 1.0 Å (Figures 4g,h). The simulation results reveal that the TAM‐TMC model exhibits a significantly increased void volume within the amorphous polymer matrix compared to TAM‐TPC, along with higher fractional free volume (FFV) values.^[^ ^19^ ^]^ Notably, the surface area of the TAM‐TMC polymer surface increased by 27.7% and showed higher microporosity than TAM‐TPC (Figure S38 and Table S3, Supporting Information). While the minimum pore sizes were similar, simulated pore size distributions confirmed that TAM‐TMC possesses a narrower distribution (Figure 4i), whereas TAM‐TPC nanofilms exhibit a larger average pore size. These computational findings are consistent with our BET analysis.^[^ ^32^ ^]^

### 2.6 Separation Performance of 3D CON Membranes

**Figure 5:**
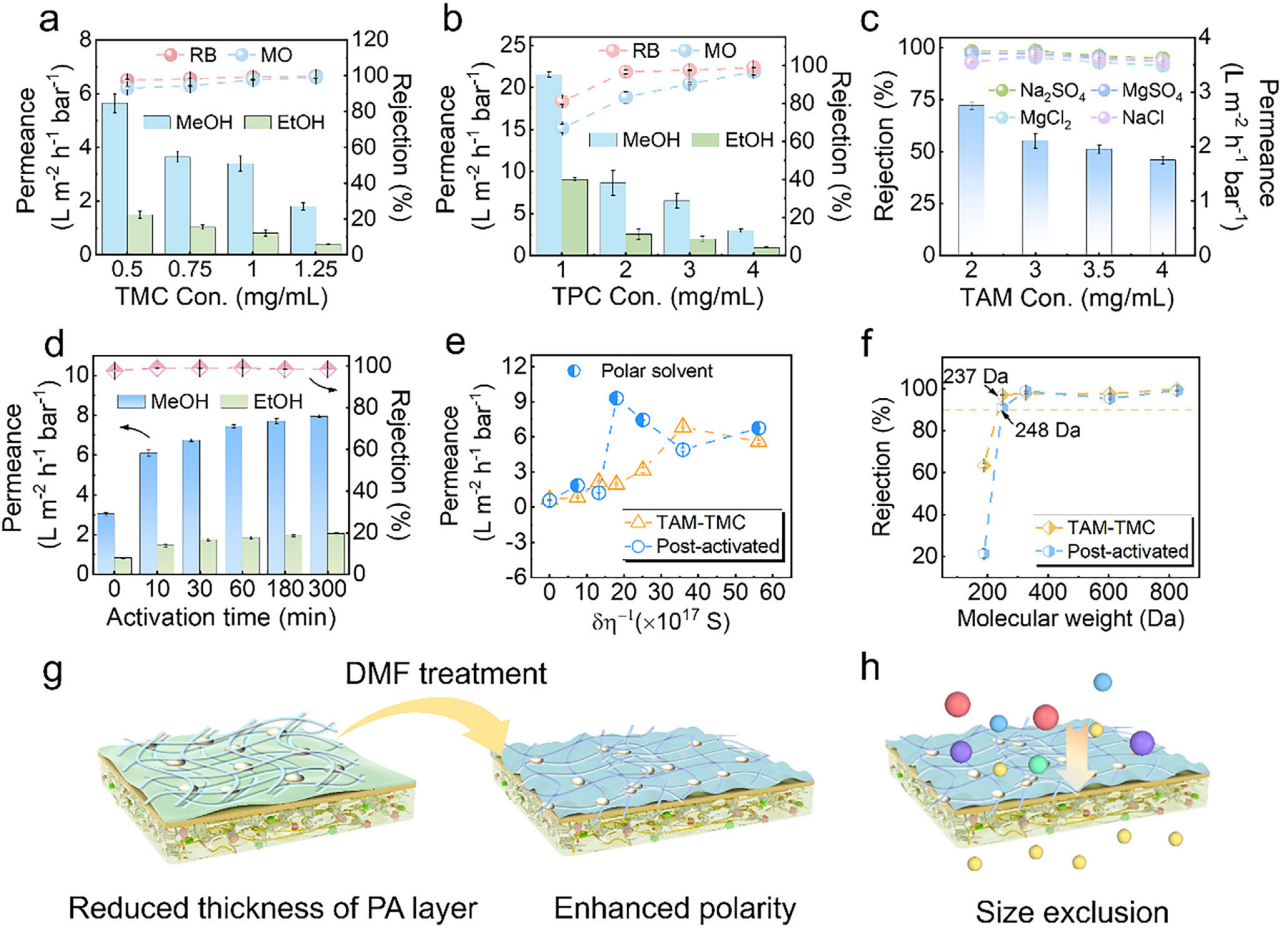
OSN separation performance of 3D CON membranes. a) The solvent permeability and rejection performance of PAH membranes prepared with different TMC concentrations and b) TPC concentrations; c) NF performance of TAM‐TMC membranes for different inorganic salts; d) separation performance of TAM‐TMC membranes with different DMF post‐treatment time; e) Relationship between the permeance of solvent through different CON membranes and solvent properties; f) Rejection of TAM‐TMC membrane before and after activation for various dyes; g) Schematic illustration of the fabrication of TAM‐TMC TFC membrane and the solvent activation process. h) Schematic diagram of molecular sieving by 3D CON membranes.

CON‐PA membranes, with their low film thickness and highly microporous characteristics, are anticipated to provide competitive solvent permeability and solute selectivity. To validate this, the impact of monomer concentrations and molar ratios on the separation performance of CON‐PA membrane was systematically studied. Our findings revealed that, at fixed TMC concentrations of 0.05 w/v% or 0.1 w/v%, elevating the TAM concentration from 0.05 to 0.25 w/v% led to a significant increase in methyl orange (MO) rejection and a moderate reduction in methanol permeance. The optimal TAM concentration was determined to be 0.2 w/v% (Figure S39, Supporting Information). Furthermore, elevating the TMC concentration from 0.05 w/v% to 0.125 w/v% resulted in an increase in MO rejection from 93.1% to 99.2% but accompanied by a decrease in methanol permeability (**Figure** **5**a). Notably, as shown in Figure S40 (Supporting Information), CON‐PA membranes exhibited a significant increase in MO rejection from 63.9% to 98.1% when increasing the concentration of TAM and TMC in equal proportions, ascribed to a substantial rise in their crosslinking degree. Here we identified the overall optimized performance for the CON‐PA membrane as 3.4 L m^−2^ h^−1^ bar^−1^ methanol permeability and 97.1% MO rejection. Figure 5b depicts the performance of TPC‐regulated CON membranes that rising TPC concentration from 0.1 w/v% to 0.4 w/v% substantially enhanced MO rejection up to 96.7% while maintaining a high methanol permeability of 3.0 L m^−2^ h^−1^ bar^−1^. To further demonstrate the effect of TAM steric hindrance on TFC membrane performance, conventional planar monomers (PIP, MPD) were selected to prepare PA membranes for comparison using identical experimental conditions. The results showed that our fabricated TAM‐TMC membranes with 3D interconnected nanochannels excel in OSN performance (Figure S41, Supporting Information).

Importantly, the resulting CON‐PA membranes demonstrated competitive performance in low‐pressure removal of monovalent and divalent salts. As exhibited in Figure 5c, the TAM_0.3_‐TMC_0.1_ membranes showed comparably high NaCl rejection of 96.7% along with a superior water permeability of 2.2 L m^−2^ h^−1^ bar^−1^. This demonstrates their excellent ion separation performance for water treatment, complementing their strong performance in organic solvents. Additionally, the TAM‐TMC membrane showed excellent adaptability across varying transmembrane pressures (Figure S42), Supporting Information. We further explored the effect of solvent polarity on membrane performance through cyclic stability tests using methanol, ethanol, and n‐hexane. The results confirmed the outstanding resistance of CON‐PA membranes across this solvent polarity gradient (Figure S43, Supporting Information).

Leveraging the established principle of solvent activation for enhancing liquid permeance in polyamide membranes, we demonstrate its profound impact on our newly developed CON‐PA TFC membranes. Following a 60 min DMF activation, the methanol permeance of activated CON‐PA membranes notably augmented up to 7.5 L m^−2^ h^−1^ bar^−1^, which is a 3.2‐fold increase in permeability to the untreated membrane. While the MO rejection largely increasing from 97.8% to 99.2%. Even with prolonged activation, the rejection performance remained uncompromised. This strongly indicates that DMF treatment precisely and selectively etched only the low‐molecular‐weight fragments, critically maintaining the integrity of the high‐crosslinked polyamide regions. The resulting exposure of active sites then drove a beneficial PA chain rearrangement, fostering the development of a more highly ordered covalent organic network.^[^ ^33^ ^]^ This insightful mechanistic understanding is robustly supported by XPS analysis, which independently corroborated an increased crosslinking density following DMF activation. Additionally, the permeability of different various solvents at a pressure difference of 1 MPa versus solvent parameters (δ) and viscosity (µ) is shown in Figure 5e. Crucially, the activated membranes exhibited a distinct shift in transport properties, showing enhanced permeability for polar solvents and reduced permeability for nonpolar solvents. This selective transport behavior was further studied by determining the molecular weight cut‐off (MWCO) using dye molecules of varying molecular weights (Table S4, Supporting Information). The MWCOs of TAM‐TMC, activated‐TAM‐TMC, and TAM‐TPC were 237, 248, and 245 Da, respectively (Figure 5f; Figures S44 and S45, Supporting Information). These close MWCO data potentially show that DMF post‐treatment effectively reduces the thickness of the selective layer in TFC membranes, thus reducing mass transfer resistance. Furthermore, we propose that the dynamic rearrangement of PA molecular chains, induced by DMF activation, leads to the exposure of polar functional groups like unreacted amino groups and amide‐bonded carbonyl groups pores (Figure 5g).^[^ ^34^ ^]^ After activation, the water permeability of the membrane increased by 3.75‐fold. Due to the larger hydrated radius of SO_4_^2−^ (≈0.38 nm) compared to Cl− (≈0.33 nm) and its divalent nature, the activated TAM‐TMC membranes with the enhanced negative charge and slightly increased MWCO, maintained higher rejection of Na_2_SO_4_ and MgSO_4_ via the synergy of electrostatic repulsion and size exclusion. In contrast, the rejection of NaCl and MgCl_2_ were observed to decrease accordingly (Figure S46, Supporting Information). DMF activation also effectively enhances the intrinsic polarity of the intrinsic nanochannels, thereby promoting the preferential transport of polar solvents. Demonstrating the practical utility of these refined membranes, the activated TAM‐TMC membrane was subsequently applied to fractionate binary mixtures of molecularly dissimilar species. The results confirmed the selective passage of azobenzene and high retention of MO molecules, underscoring the high potential of using activated 3D‐TAM‐TMC membranes for fractionating the binary dye/intermediate mixtures (Figure 5h; Figure S47, Supporting Information).

### 2.7 Concentration of High‐Value‐Added Drugs

**Figure 6:**
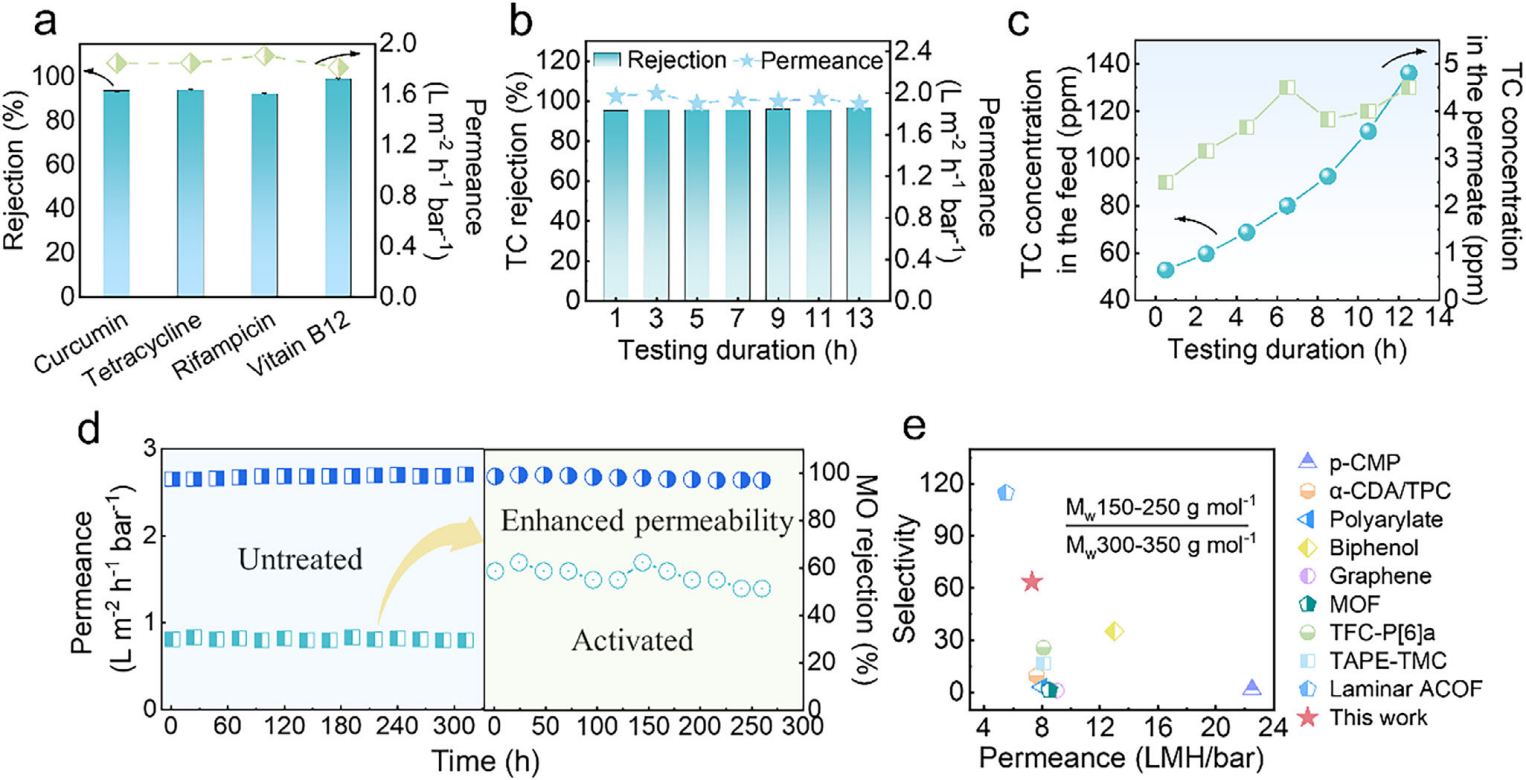
Activated TAM‐TMC membranes for precise molecular separation. a) Comparison of separation performance of activated TAM‐TMC membranes for curcumin, tetracycline, rifampicin, and vitamin B12; b,c) Separation performance of the activated TAM‐TMC membrane during concentration of 50 ppm of TC in EtOH; d) Long‐term operational stability performance of activated TAM‐TMC membrane before and after activation; e) Performance comparison in methanol permeance and selectivity between solutes with molecular weights of 100–250 over 300–350 g mol^−1^.^[^ ^4^, ^10^, ^12^, ^28^, ^31^, ^35^ ^]^

To evaluate the practical applicability of CON‐PA membranes, DMF‐activated TFC membranes were utilized for the separation of structurally and functionally diverse antibiotics (specifications in Table S5), Supporting Information, demonstrating their high potential for active pharmaceutical ingredient (API) enrichment. The activated TAM‐TMC consistently showed exceptional rejection, surpassing 92% across a panel of challenging molecules, including tetracycline, chlorophyll, rifampicin, and vitamin B12 (Figure **6**a). Crucially for practical implementation, the TAM‐TMC also demonstrated exceptional operational stability over a 13 h continuous cross‐flow test with tetracycline in ethanol at 10 bar (Figure 6b). These robust and sustained performance metrics demonstrate that activated TAM‐TMC membranes achieve superior tetracycline rejection, exceeding 95.4%, while simultaneously maintaining a substantial ethanol permeability of ≈2 L m^−2^ h^−1^ bar^−1^. Extended operation for 13 h under continuous concentration conditions demonstrated the robust performance of CON‐PA membranes, achieving a final tetracycline (TC) concentration of 136.2 ppm, representing a 2.6‐fold enrichment from the initial feed (Figure 6c). Analogous favorable operational stability was also maintained during continuous tests with vitamin B12 (Figures S48 and S49, Supporting Information). These compelling results underscore the high potential of CON‐PA membranes for pharmaceutical concentration applications.

A paramount consideration for real‐world implementation is long‐term operational stability. To this end, we performed extensive continuous tests on TAM‐TMC and activated TAM‐TMC membranes by evaluating the ethanol solvent permeability and MO rejection. The activated membranes demonstrated a stable ethanol permeability of 1.8 L m^−2^ h^−1^ bar^−1^, a marked improvement over the 0.8 L m^−2^ h^−1^ bar^−1^ observed for unmodified TAM‐TMC. Concurrently, MO rejection consistently remained above 98% throughout testing, with both pristine and activated membranes exhibiting outstanding operational stability, which confirms the integrity of the PA layer (Figure 6d). Figure S50 (Supporting Information) further highlights the operational stability of our fabricated 3D‐CON membranes, which maintained stable and excellent filtration performance over 260 h. To contextualize our membranes performance within the broader OSN, we correlated the MWCOs of the three PA membranes with their methanol permeability (Figure S51, Supporting Information). Results reveal that the activated TAM‐TMC membranes demonstrate superior selectivity for small‐molecule dyes compared to most of the previously reported OSN membranes (Figure 6e; Table S6, Supporting Information), rendering them highly competitive for potential applications in organic solvent separations.

## 3 Conclusion

This work introduces an innovative chemically confined crosslinking strategy to construct sophisticated 3D regionally ordered polyamide networks. CON‐PA membranes were in situ fabricated on porous Kevlar hydrogel by leveraging tetrahedral TAM and acyl chloride monomers. A key innovation lies in the acid‐induced cconfined IP reaction, which precisely modulates the amide reaction and facilitates the controlled alignment of PA chains into semi‐crystalline CON‐PA. Both experimental and simulation result demonstrate that varying the number of chloride groups in the monomer significantly enhances free volume and precisely narrows the pore size distribution within the nanofilms. This structural control culminates in the formation of interconnected sub‐nanochannels featuring a hierarchical 3D porous architecture within the PA layer. Our systematic investigation of 3D‐CON membranes, prepared via concentration‐modulated IP reaction, profoundly illustrates how subtle shifts in monomer concentration critically dictate both membrane structure and OSN performance. Moreover, DMF activation of TAM‐TMC membranes offers a feasible approach to effectively alleviate detrimental over‐crosslinking and optimize the selective layer thickness, enabling unprecedented rapid solvent transfer and highly efficient molecular separation. The activated membranes exhibited excellent methanol permeability up to 7.5 L m^−2^ h^−1^ bar^−1^ and MWCO of 248 Da, which exceeded the performance of most conventional PA‐OSN membranes. Importantly, precise fractionation of mixed dyes in organic solvent systems is achievable, highlighting the outstanding application potential of these membranes for antibiotic concentration. This study introduces a robust separation system based on 3D CON membranes, enabling efficient and precise separation of molecular‐scale substances in demanding organic solvent environments.

## Conflict of Interest

The authors declare no conflict of interest.

## Author Contributions

All the authors contributed to this article.

## Supporting information



Supporting Information

## Data Availability

The data that support the findings of this study are available on request from the corresponding author. The data are not publicly available due to privacy or ethical restrictions.
