# Supplementary material for: 3D Covalent Organic Network Membranes With Regionally Ordered Nanochannels for Efficient Molecular Sieving
Source: Adv Sci (Weinh). 2025 Aug 4;12(40):e10911. doi: 10.1002/advs.202510911 (PMC12561272; doi:10.1002/advs.202510911)
Supplement: Supplementary file 1 — Supporting Information [file ADVS-12-e10911-s001.docx]

Supporting Information

**Three-dimentional covalent organic network membranes with regionally ordered nanochannels for efficient molecular sieving**

Lei Ge , Gaojing Du , Hengjie Song , Jiaqi Li , Jingwei Hou , Yatao Zhang *, and Junyong Zhu *

Lei G, G. Du, H. Song, J. Li, Y. Zhang, and J. Zhu

School of Chemical Engineering

Zhengzhou University

Zhengzhou 450001, PR China

Emails: zhangyatao@zzu.edu.cn; zhujunyong@zzu.edu.cn

J. Hou

School of Chemical Engineering

The University of Queensland

St Lucia, QLD 4072 (Australia)

**1. Experimental**

*1.1 Materials and chemicals*

Kevlar aramid fibers (linear density: 220 dtex; break strength: 49 N; modulus: 3.000 cN/tex) were obtained from Thread Exchange (Delaware, USA). The polypropylene/polyethylene (PP/PE) non-woven fabric (Novatexx 2471) was provided by Freudenberg Performance Materials (Weinheim, Germany). Potassium hydroxide (85.0%), dimethylsulfoxide (DMSO, 99.5%), and dichloromethane (DCM)， dioxane, tetrahydrofuran (THF), and N,N-dimethylformamide (DMF), methanol (MeOH), ethanol (EtOH), acetonitrile, tetrahydrofuran (THF), and n-hexane were acquired from Sinopharm Chemical Reagent (Shanghai, China). Acetone were obtained from Luoyang Chemical Reagent Factory. Tetrakis(4-aminophenyl)methane (TAM, >98.0%) were purchased from Shanghai Tengqian Biological Technology Co., Ltd. (Shanghai, China), Trimesoyl chloride (TMC, 98.0%) and Terephthaloyl chloride (TPC, 98.0%) were purchased from TCI (Shanghai, China). Deionized water was used throughout the experiment unless specified. Azobenzene (AZB, 188.2 g/mol), Basic Orange 2 (BO2, 248.7 g/mol), methyl orange (MO, 327 g/mol), acid red 27 (AR, 604.47 g/mol), brilliant blue (BBR, 826 g/mol), rose Bengal (RB, 1017 g/mol), curcumin (368.38 g/mol), tetracycline (TC, 444.34 g/mol) and Rifampicin (822.94 g/mol) were supplied by Aladdin Co. Ltd. (Shanghai, China). VB12 (1355.37 g/mol) was obtained from Macklin Reagent Co., Ltd. (Shanghai, China).

*1.2* *Characterization*

The surface morphology and cross-sectional image of the membrane was visualized using a Focused ion beam scanning electron microscopy (FSEM) (Zeiss). The surface roughness, morphology of the TAM-TMC membrane and TAM-TPC membranes were analyzed using an Atomic Force Microscope (AFM) (Bruker Dimension Fast Scan). High-resolution transmission electron microscopy (HR-TEM, JEOL JEM-F200) and energy-dispersive spectrometry (EDS) was used to characterize the morphologies of free-standing nanofilms and the thickness of the selective layer for TFC membranes.

The TEM specimens were made by immersing them in epoxy resin and then passing them through a microtome. Attenuated total reflectancefourier transform infrared spectroscopy Fourier-transform infrared spectroscopy (ATR-FTIR) (Nicolet 560) was applied to analyze the chemical structures of the monomer and nanofilms using the KBr pellets method. X-ray photoelectron spectroscopy (XPS, Thermo Fisher, Kɑ line) was used to probe the surface chemical compositions of TFC membranes. Power X-ray diffraction (XRD) patterns of TAM-TMC/TPC membrane samples (PANalytical, the Netherlands) were analyzed with a scanning range of 2θ from 2° to 50°. The Zeta potential of the membrane was determined using a SurPASS™ 3 electrokinetic analyzer (Anton Paar surpass3) in a 1mM potassium chloride solution over a pH range of 3 to 10. TGA analysis was conducted using a TG209F3 Tarsus instrument (TGA, Netzsch, Germany) under nitrogen (N_2_) gas atmosphere with the temperature ranging from 20-800°C and a heating rate of 10°C min^-1^. To obtain the hydrophilicity of membranes, the different solvent contact angle was measured using a contact angle instrument (OCA25, Dataphysics instruments, Germany), wherein 5 μl of solvent droplets at three different locations was recorded to get the average data. The mechanical properties of the membrane were evaluated using an Electronic Universal Testing Machine (China Meitersi Industrial System CMT6103). The N_2_ adsorption isotherm and specific surface area of the polyimine membrane were calculated using the Brunauer-Emmett-Teller method (Quantachrome Autosorb IQ), and the pore size distribution of the membrane was determined through density functional theory calculations. The GIWAXS (Xeuss 2.0) measurements of the nanofilms were performed on a Xenocs Xeuss 3.0. Before measurement, the nanofilms samples were transferred onto a silicon substrate and dried, and all samples were placed under a vacuum to eliminate atmospheric scatter. Liquid Nuclear Magnetic Resonance Spectroscopy (Bruker-Avance NEO Ascend 500M) for evaluating diffusion coefficients of aqueous monomers.

*1.3 Preparation of Kevlar substrates*

The preparation of the Kevlar hydrogel-supported membrane was fabricated based on the method reported in our previous work, following the principle of wet phase inversion.^[1]^ The specific procedure is as follows: First, potassium hydroxide (KOH, 1.2 g) was weighed and dissolved in of deionized water (1.2 mL) using ultrasonic treatment in the bottle. Then the Kevlar fiber (1.2 g) and DMSO (60 mL) were added to the bottle and magnetically stirred in a water bath (30-40 ℃) for 48 h. The resulting deep red solution was centrifuged at 8000 rpm for 30 minutes to remove bubbles and KOH precipitates. Next, The PP/PE non-woven fabric was fixed onto a clean glass plate, and the Kevlar casting solution was spread onto the nonwoven substrate using a 250 μm thick doctor blade. The support layer was immersed in aqueous solution at 25 ℃ water bath, where phase inversion occurred in deionized water for 6 h to produce a Kevlar hydrogel carrier membrane, and then soaked overnight in deionized water to remove residual solvent before use. The prepared Kevlar hydrogel support exhibited a water permeation flux of 600-700 LMH bar^-1^.

*1.4 Preparation of free-standing 3D CON nanofilms*

Under ambient temperature and atmospheric pressure, the same volume of aqueous hydrochloric acid solution of TAM (0.2 w/v%) and hexane solution of TMC (0.1 w/v%) were configured as aqueous and organic phases, respectively. Free-standing nanofilms were fabricated via IP at the water-hexane interface. After 3 minutes of reaction, the resulting nanofilms were collected and sequentially washed with deionized water and ethanol. The obtained TAM-TMC/TPC nanofilms were subsequently transferred onto various substrates for comprehensive characterization.

*1.5 Interfacial diffusivity of TAM monomers*

TAM was dissolved in water and HCl aqueous solution, respectively, and the absorbance at 190-400 nm was tested. The standard curve was determined by scanning the full spectra of a series of TAM aqueous solutions in the range of 1-5 mg/L. The wavelength of 210 nm was selected as the detection wavelength and the linear equation was determined from the absorbance values corresponding to different concentrations of TAM aqueous solutions. Subsequently, 2.2 mL of TAM (or TAM+HCl) aqueous solution and 1.2 mL of n-hexane were added to a quartz cuvette to construct a liquid-liquid interface. The solution volumes were carefully controlled to be identical, with the aqueous phase surface positioned above the UV beam point. The monomer concentration at different diffusion times was periodically monitored.^[2]^

*1.6 Calculation of relative crystallinity of TAM-TMC nanofilms*

The relative crystallinity was then calculated as the ratio of the crystalline peak area to the sum of the crystalline peak area and the scattering peak area.^[3]^ The calculation formula is as follows:

$$\varepsilon= \frac{Ic}{Ic+Ia} \times100\%$$

where *Ic* is the integrated intensity of the crystalline peak, *Ia* is the integrated intensity of the amorphous peak.

*1.7 Fabrication of 3D CON membranes*

The TAM-TMC/TPC composite membrane was fabricated on a Kevlar hydrogel-supported membrane via amidation between acyl chloride functionalities and amine monomers, as illustrated in Figure S1. Firstly, residual liquid on both the surface and backside of the Kevlar hydrogel was removed by nitrogen purging. Subsequently, the front side of the Kevlar substrate was exposed to an aqueous TAM solution (10 mL, 0.05-0.25 w/v%) containing 17 Mm HCl for 5 minutes. After removing the aqueous solution, the surface of TAM-loaded hydrogel was exposed to hexane solution of TMC (0.05-0.25 w/v%) to initiate the IP process and construct a PA selective layer. The membrane was then placed in a 70 °C oven for thermal crosslinking for 3 minutes. The prepared membranes were stored in deionized water prior to use. Error bars in presented data obtained from three independently fabricated membranes represent the standard measurements deviation.

*1.8 Post-activation treatment of 3D CON membranes*

According to the method in the literature of Li et al., the TFC membranes prepared after thermal cross-linking were immersed in DMF for different times (10 min, 30 min, 1 h, 3 h, 5 h) and then stored in methanol for 1 h.^[4]^ The membranes were rinsed with DI water before testing.

*1.9 Mechanical stability performance testing*

Turn on the power of the electronic universal tester and running up for 15 minutes. Open the test software and set up the experimental method (Test Mode: Single Test; Test Type: Tension; Shape: Sheet; Speed: 1 mm/min). Measure the thickness and width of the sample using calipers and input these measurements into the software. Determine the gauge length based on the sample length and save the experiment file. Click on "Calibration." Place the sample into the fixture, zero the displacement and force values, and then click "Start" to begin the test. After the final test, the exported Excel sheet contains three columns of data: time, load, and displacement.

Stress = Load/ (Thickness * Width)

Strain=L/L_0_*100%

Where L_0_ is the initial gauge length of the sample, and ΔL is the deformation length of the sample under load (equivalent to the displacement or travel).

Young's modulus: Determine Young's modulus by selecting a linear segment (set the loads at the ends of the linear segment to predefined points

Young's modulus = slope * gauge length /sample area

*1.10 Calculation of membrane surface crosslinking degree*

The O/N in the polyamide membrane was obtained by XPS analysis, and the degree of crosslinking (C, %) can be calculated further using equations (4) and (5).^[5]^


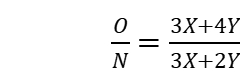


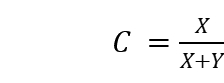


Where X and Y represent the network crosslinked and linearly crosslinked structures of the polyamide layer, respectively.

*1.11* *OSN performance tests*

The performance of the OSN membrane was evaluated using pure solvent permeability and dye molecule rejection. Dyes with varying molecular weight dissolved in ethanol were used to study solute rejection performance (50 ppm). The fabricated membrane was installed into stainless-steel dead-end filtration cell with an effective membrane area of 4.91 cm². Prior to the filtration tests, the membrane was pre-pressurized at 1 MPa for 1 hour to stabilize the membrane. The permeability of the membrane (P, L m^-^² h^-^¹ bar^-^¹) was calculated using the following equation:


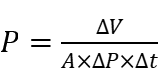


Where ∆V, A (m^2^), ∆P, Δt (h) represent the volume of the collected permeate (L), effective separation area, transmembrane pressure (bar), and actual filtration time (h), respectively.

The rejection rates (R, %) was calculated using the equation:


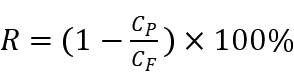


where C_P_ and C_F_ are the dyes/antibiotic concentrations (mg L^-1^) in the feed and filtrate, respectively. Analysis of concentrations was determined from UV-vis spectrophotometer (NanoDrop 2000c, Thermo Scientific) and a conductivity meter (Thermo Scientific Orion Star A212).

The selectivity (α) of the membrane for antibiotic in the antibiotic mixed system was calculated using Equation (8) by combining 50 ppm antibiotic solution in equal volumes as the feed solution:


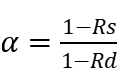


*1.12 Stability test of TAM-TMC/Kevlar membrane*

The long-term stability of the prepared membrane was evaluated by the dead-end filtration process over 100 h. Model solution (MO concentration: 50 ppm) was as feed under the pressure of 10 bar. The separation performance of MeOH or EtOH permeance and MO rejection were measured according the methods previously mentioned.

**2 Molecular dynamics (MD) simulation**

MD simulation was performed using the Gromacs 2020.6 program , using periodic boundary conditions in the XYZ direction under NPT ensemble conditions.^[6]^ In the MD simulation process, all hydrogen bonds are related to the LINCS algorithm.^[7]^ The integration step is 1 fs. The electrostatic interaction adopts the (Particle-mesh Ewald) PME method calculation. The non-bond interaction cut-off is set to 12.5 Å and is updated every 10 steps. With V-rescale the simulated temperature controlled by temperature coupling method is 298.15 K; The simulated pressure of 1.01325 bar was controlled by Parrinello-Rahman pressure coupling method. The whole simulation is divided into two systems. For system one, 50 TAM-TMCs are used to construct. For system 2, 50 TAM-TPCs are used to construct, and both TAM-TMC and TAM-TPC adopt GROMOS96 G54A7 force field.^[8]^ After constructing the initial model of the system, firstly, the energy of the system is minimized by the steepest descent method to eliminate the too close contact between atoms. Then, the equilibrium configuration was extracted after a 10-ns MD simulation performed at 298.15 K and 1 atm (1.01325 bar). For pore distribution and void analysis, Poreblazer was used for analysis, and the probe size was set to 1 Å. The free volume fraction is calculated using the following formula:


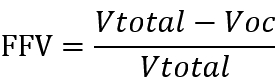


Where *Vtotal* is the final volume obtained after NPT simulation, contributed by all atoms of the polymer occupying the volume with the free volume; *Voc* is the volume occupied by the polymer.

3. Supplementary Figure


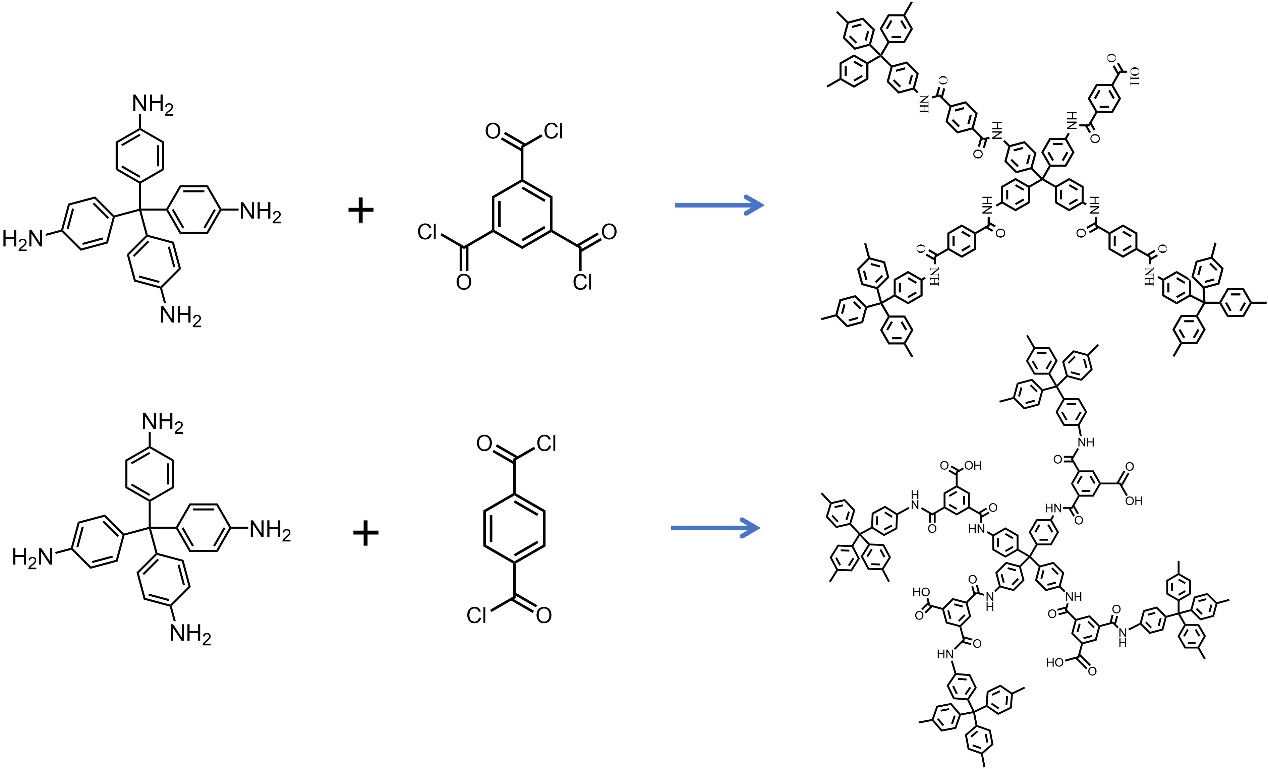


**Figure S1.** The amine monomer forms a polyamide network with two different acyl chlorines.


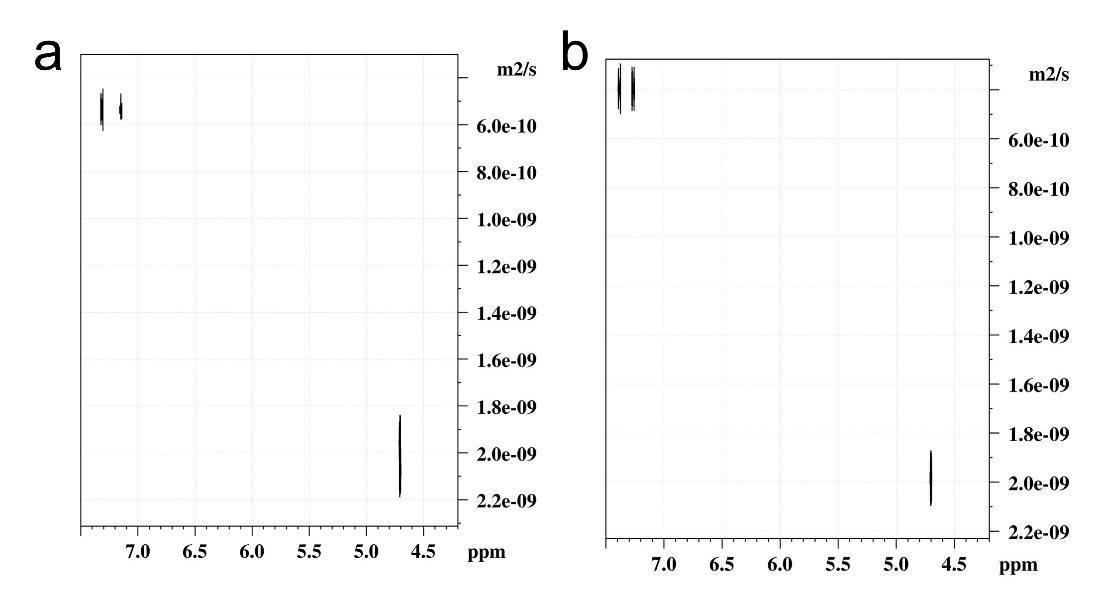


**Figure S2.** The 2D DOSY NMR spectra of TAM and TAM+HCl in D_2_O.


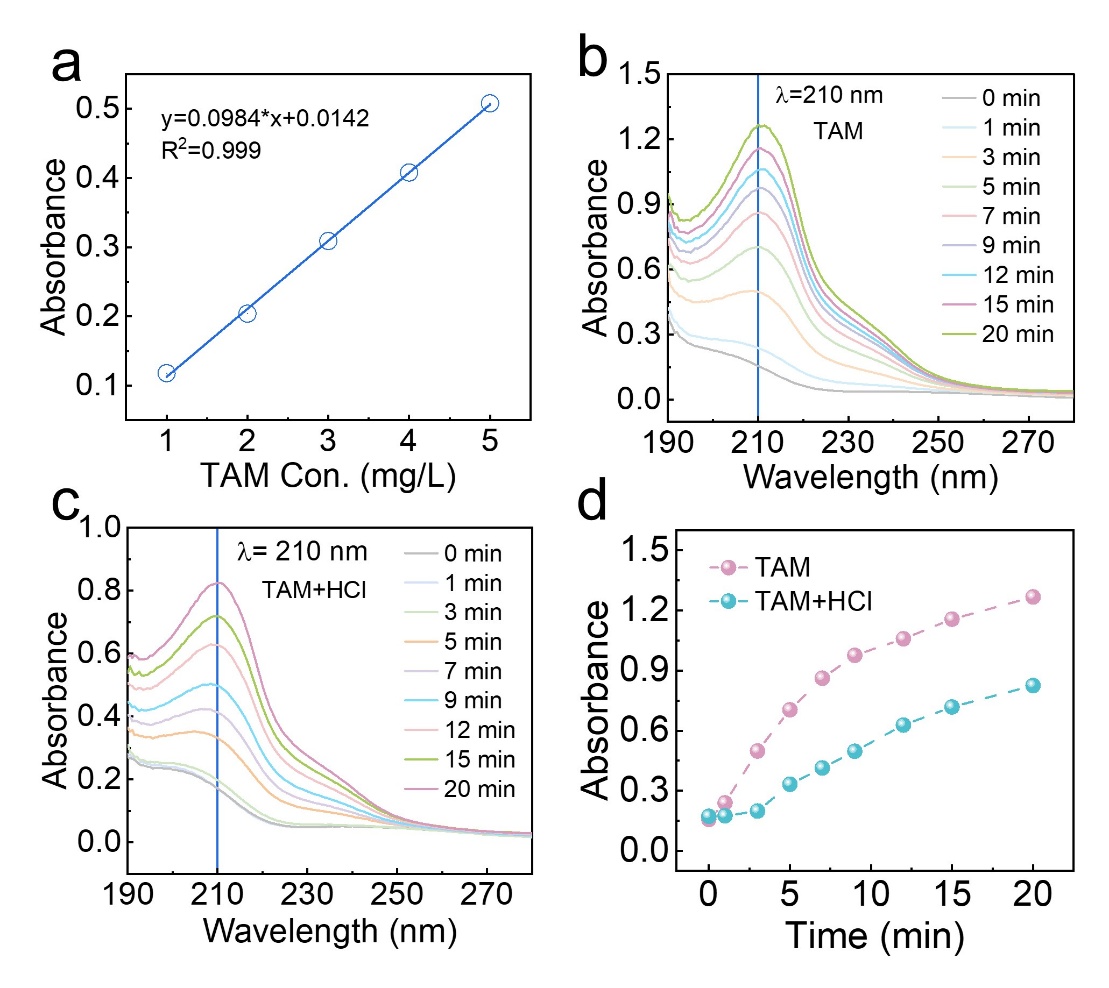


**Figure S3.** (a) Standard curve between the absorbance at 210 nm and the concentration of TAM solution. (b) UV-vis spectra of TAM in water-n-Hexane system. (c) UV-vis spectra of TAM+HCl in water-n-Hexane system. (d) UV-vis absorbance of the aqueous solution over reaction time of the interfacial polymerization.


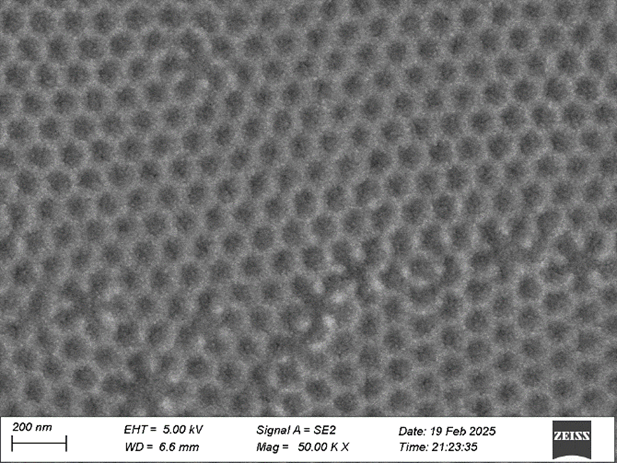


**Figure S4.** SEM images of TAM-TMC free-standing membranes.


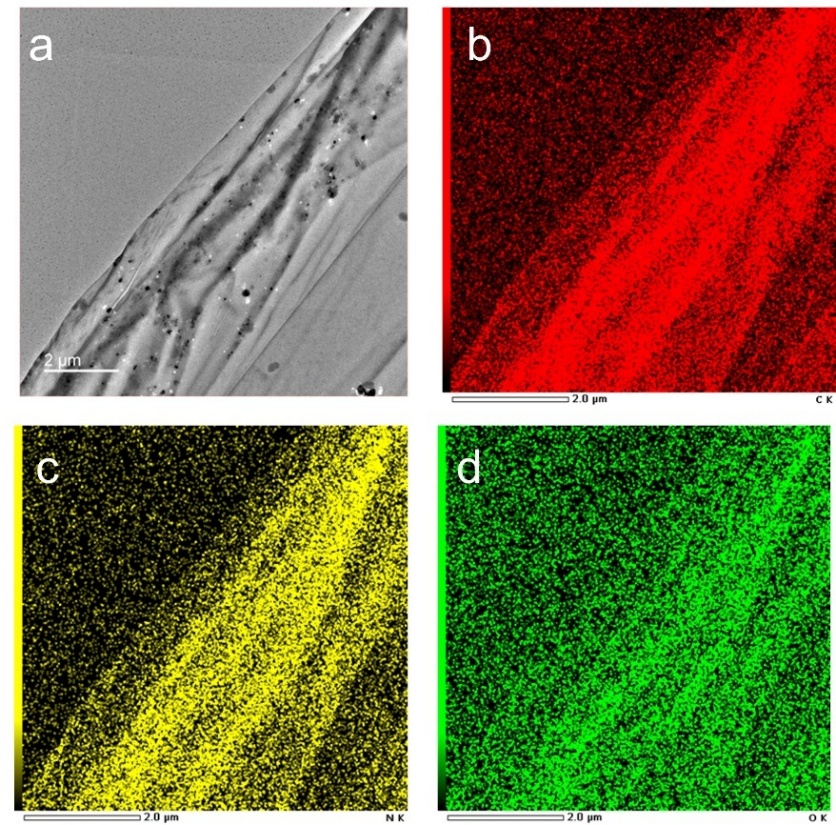


**Figure S5.** TEM/EDS mapping of the surface-section and elemental distributions of TAM-TMC free-standing nanofilms.


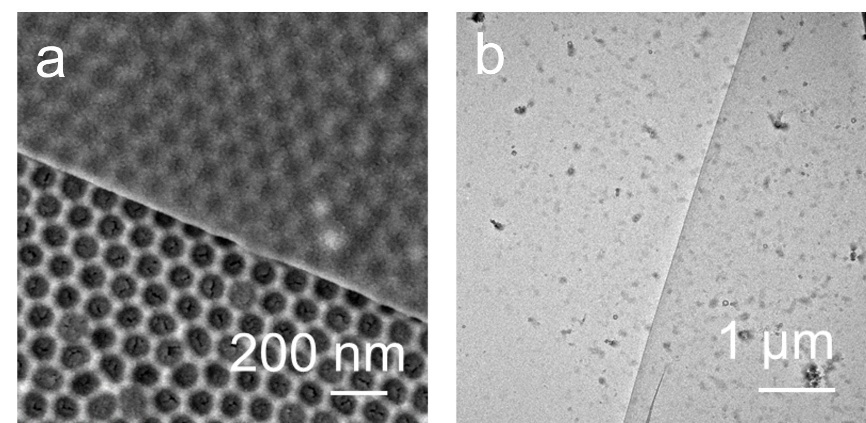


**Figure S6.** SEM images(a) and TEM images(b) of TAM-TPC free-standing nanofilms.


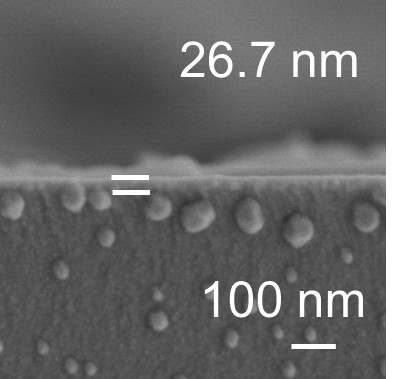


**Figure S7.** The cross-section images of TAM-TPC free-standing nanofilms.


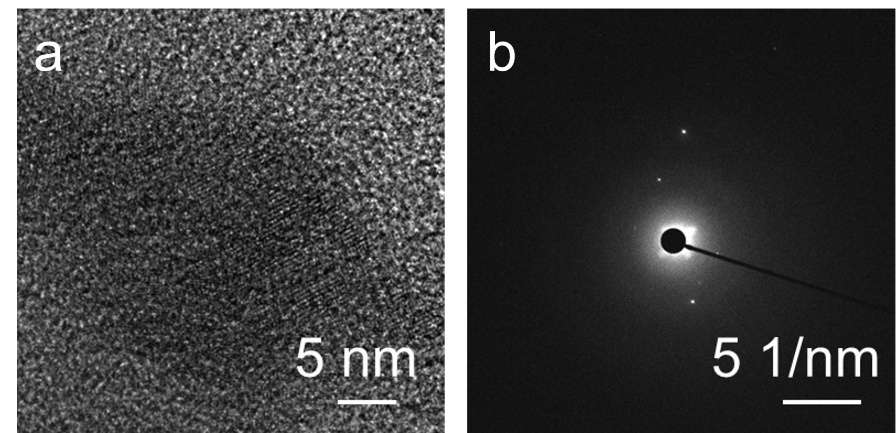


**Figure S8.** High resolution TEM images and corresponding electron diffraction of TAM-TPC nanofilms.


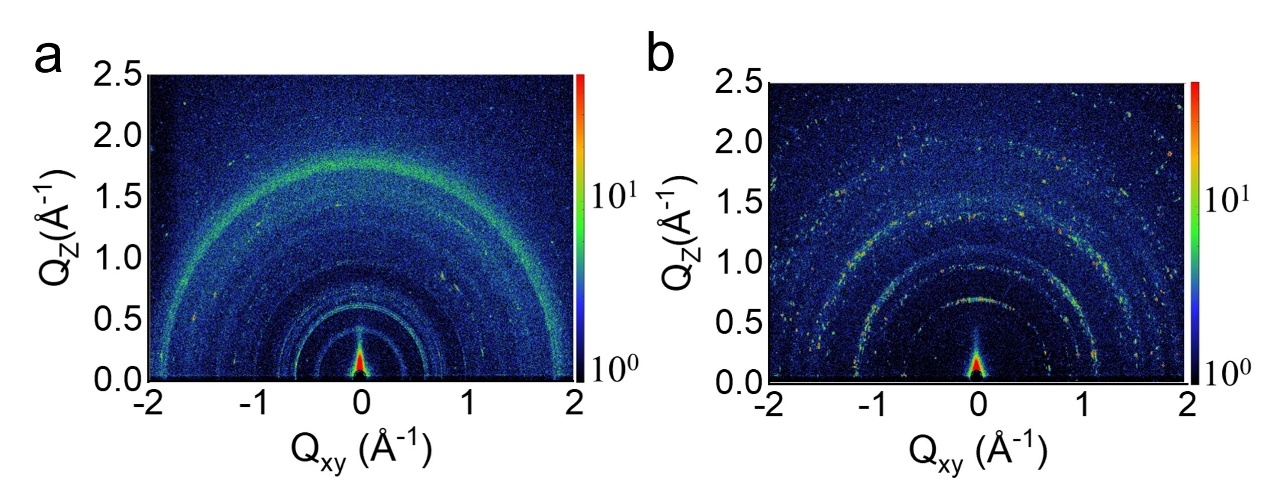


**Figure S9.** GIWAXS two-dimensional images of the (a) TAM-TMC nanofilm and (b) TAM-TPC nanofilms.


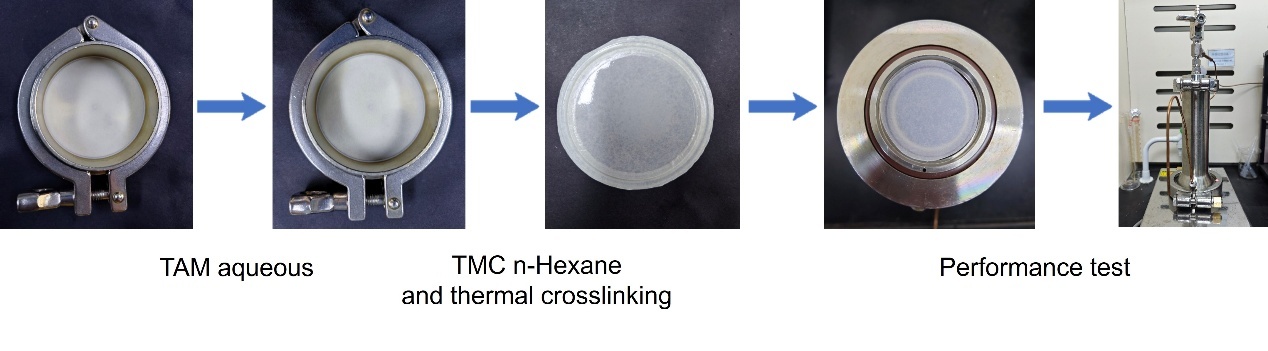


**Figure S10.** Physical diagram of the process for preparing and test TMC membranes.


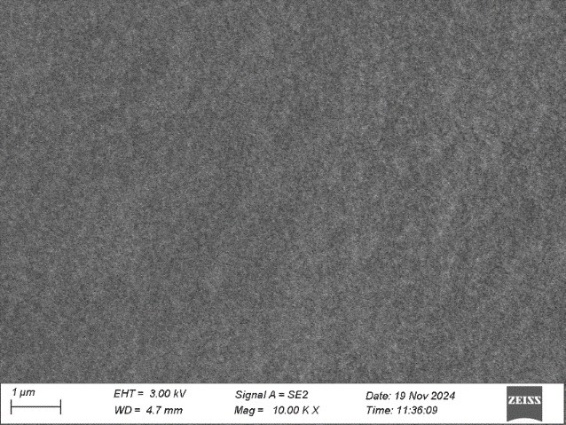


**Figure S11.** Top-view SEM images of Kevlar membrane.


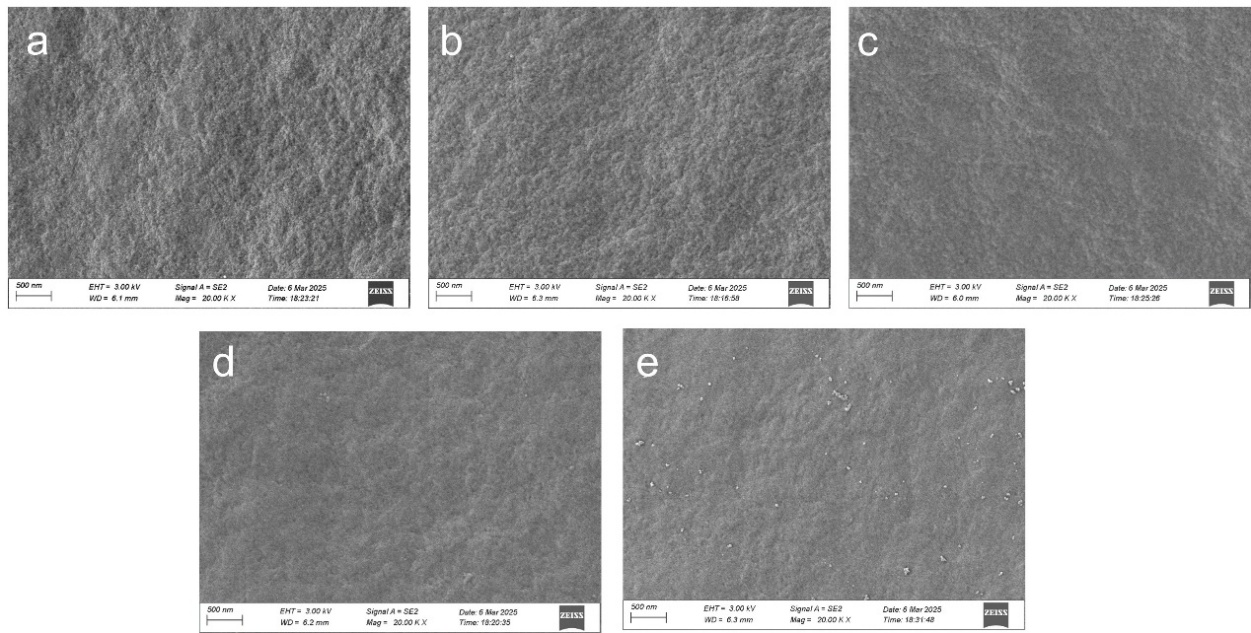


**Figure S12.** Top-view SEM images of the TAM-TMC membranes with different TAM concentrations at a fixed TMC concentration of 0.05 w/v%. (a-e) 0.05, 0.1, 0.15, 0.2, 0.25 w/v%.


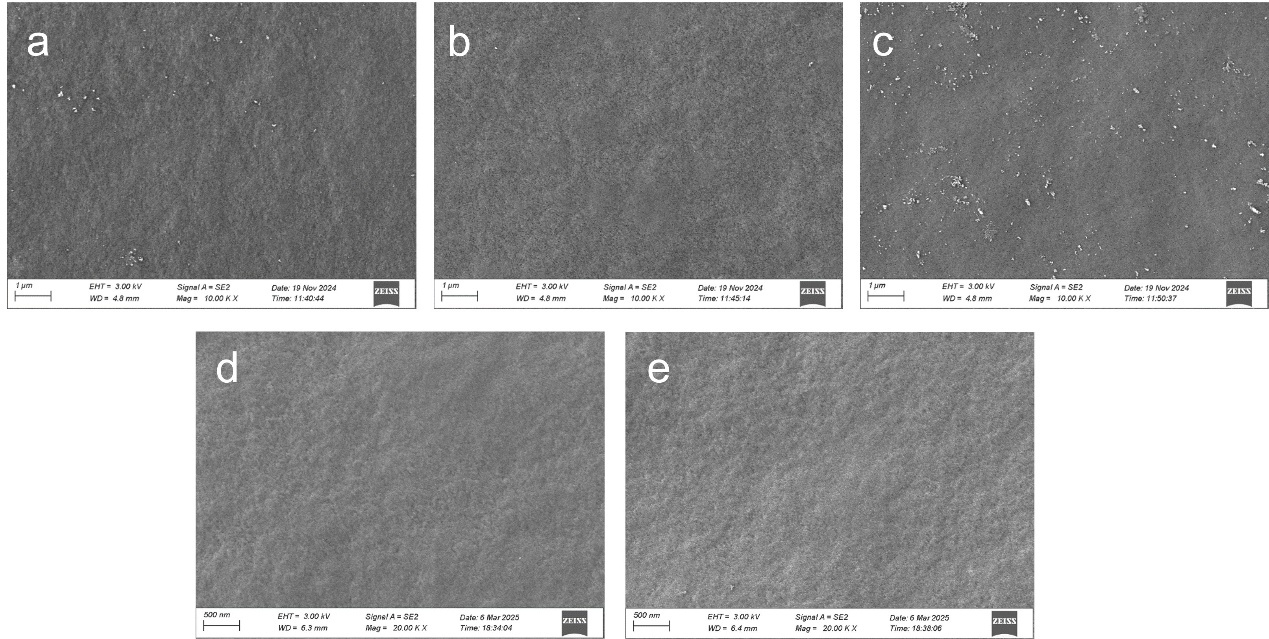


**Figure S13.** Top-view SEM images of the TAM-TMC membranes with different TAM concentrations at a fixed TMC concentration of 0.1 w/v%. (a-e) 0.05, 0.1, 0.15, 0.2, 0.25 w/v%.


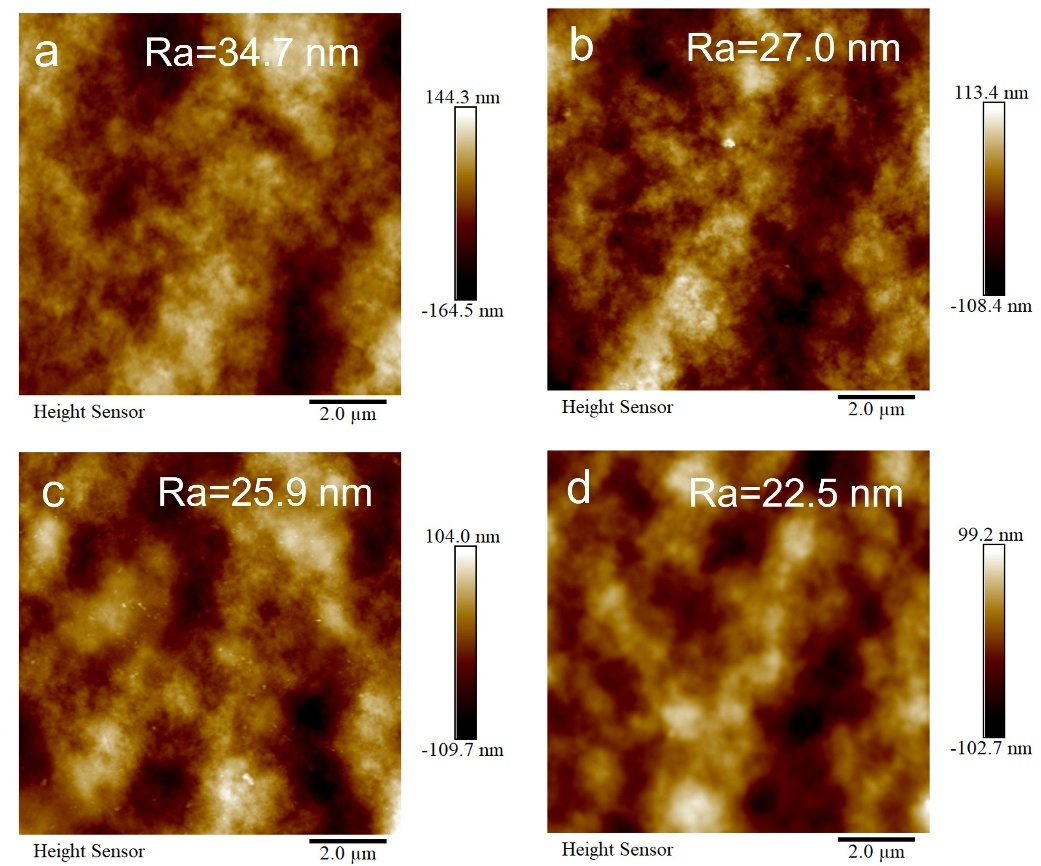


**Figure S14.** AFM images of the TAM-TMC membranes with different TAM concentrations at a fixed TMC concentration of 0.1 w/v%. (a-d) 0.05, 0.1, 0.15, 0.25 w/v%.


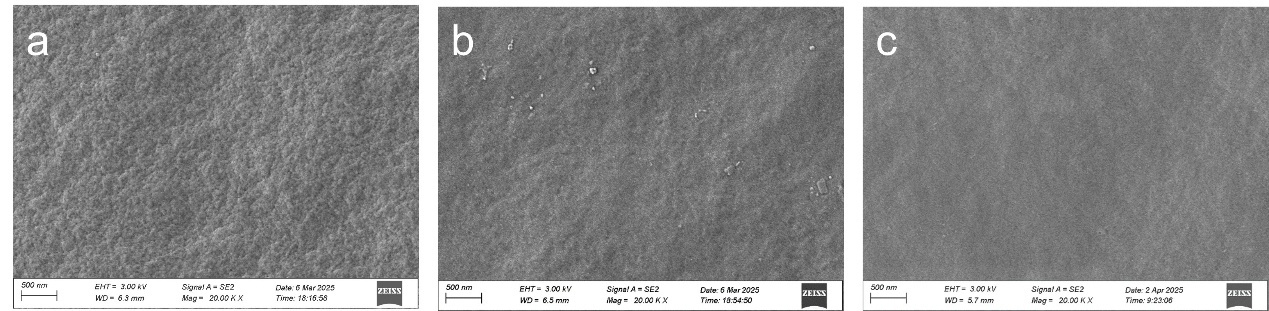


**Figure S15.** Top-view SEM images of the TAM-TMC membranes with different TMC concentrations at a fixed TAM concentration of 0.2 w/v%. (a-c) 0.05, 0.075, 0.125 w/v%.


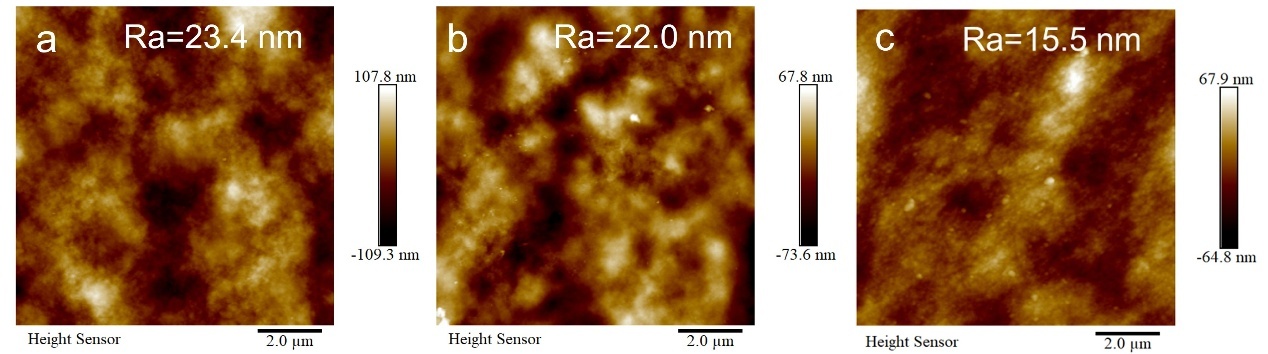


**Figure S16.** AFM images of the TAM-TMC membranes with different TMC concentrations at a fixed TAM concentration of 0.2 w/v%. (a-c) 0.05, 0.075, 0.125 w/v%.


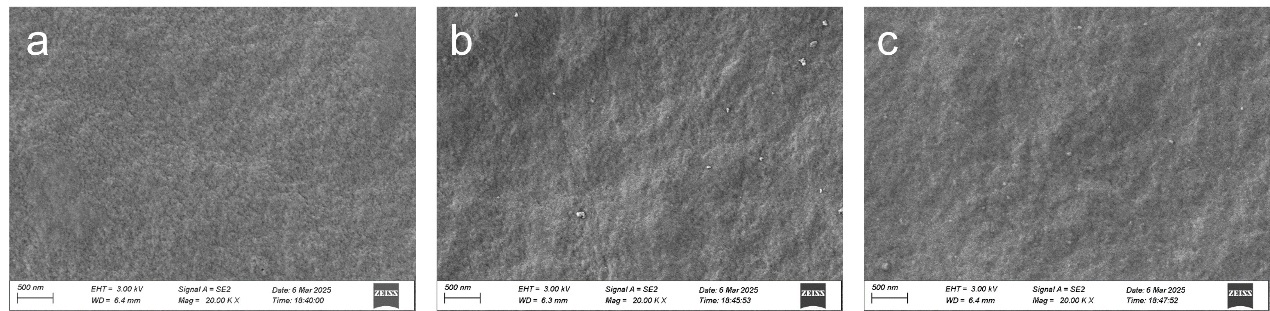


**Figure S17.** Characterizations of PA membranes. Representative SEM images of the top surface of PA membranes at different conditions. The concentration ratio of TAM to TMC (a) 0.15:0.075 w/v%, (b) 0.18:0.09 w/v%, and (c) 0.22:0.11 w/v%.


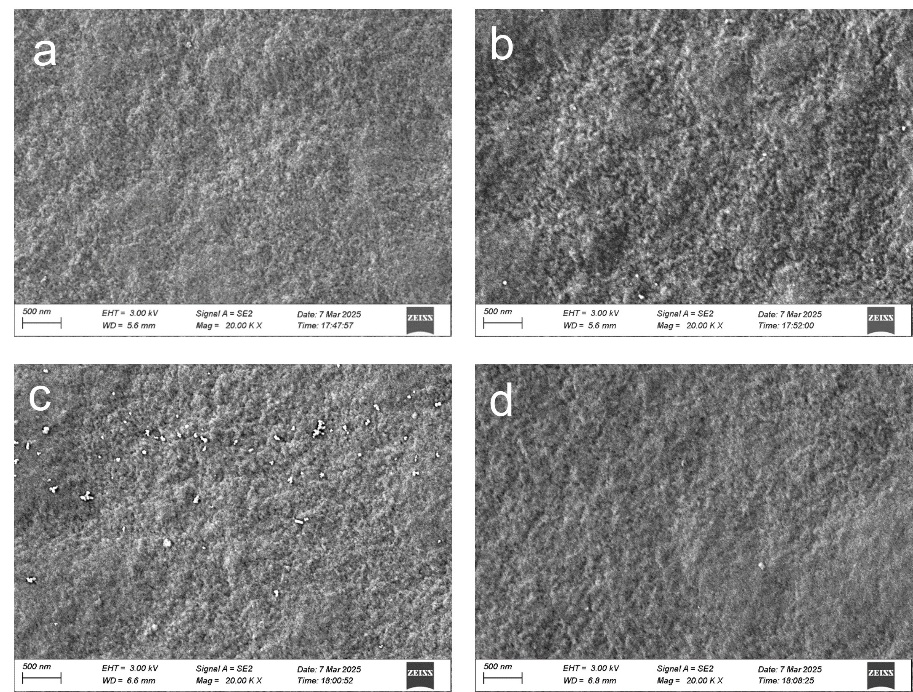


**Figure S18.** Top-view SEM images of the TAM-TPC membranes with different TPC concentrations at a fixed TAM concentration of 0.2 w/v%. (a-d) 0.1, 0.2, 0.3, 0.4 w/v%.


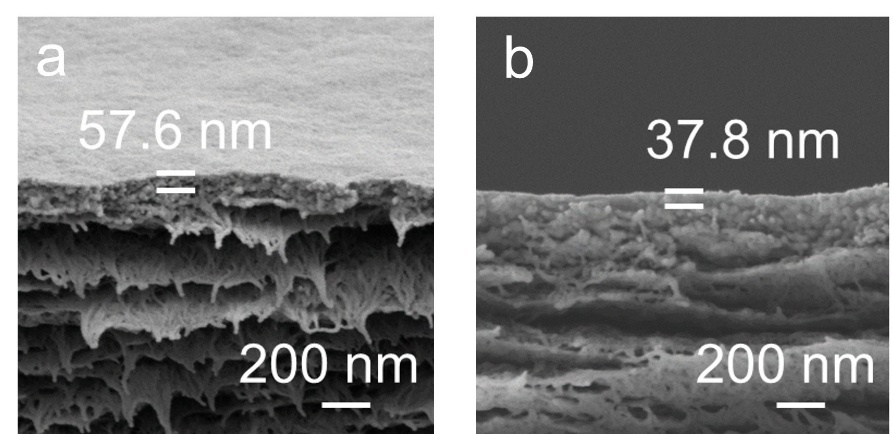


**Figure S19.** Cross-sectional SEM image of (a) TAM-TMC membranes and (b) TAM-TPC membranes.


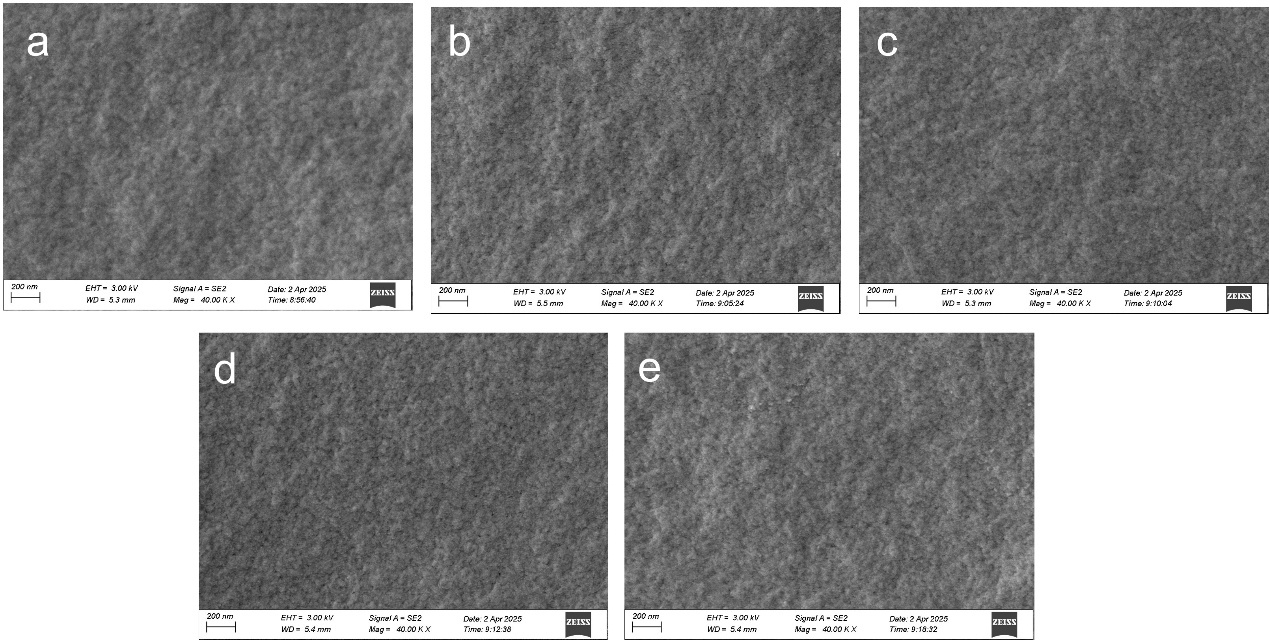


**Figure S20.** The effect of solvent treatment on morphology of TAM-TMC membrane surface. (a-e) DMF treatment time: 10 min, 30 min, 1 h, 3 h, 5 h.


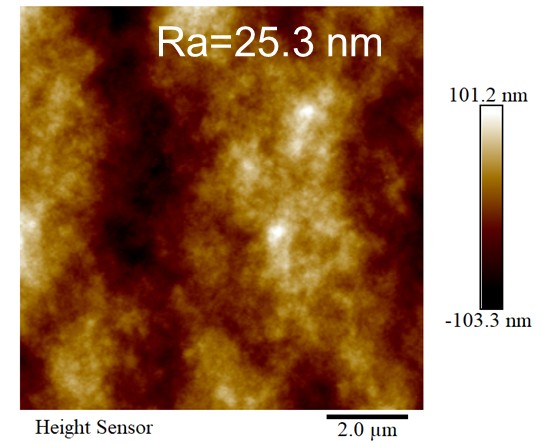


**Figure S21.** AFM image of the TAM-TMC membranes with DMF treatment 1h.


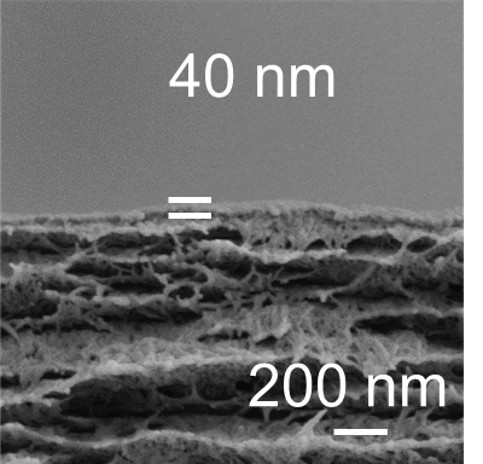


**Figure S22.** Cross-sectional SEM image of TAM-TMC membranes with DMF treatment 1h.


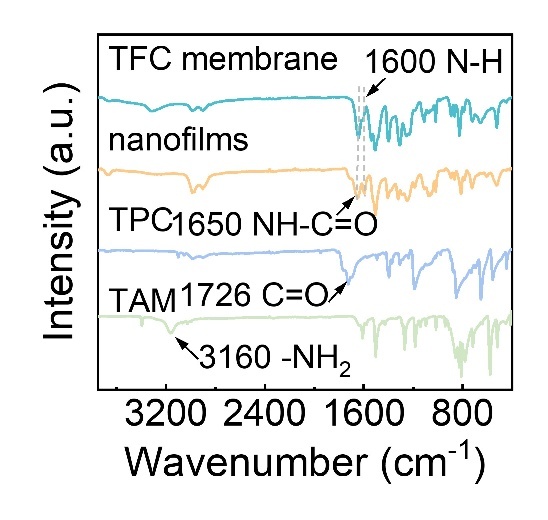


**Figure S23.** FT-IR spectrums of TAM-TPC membranes.


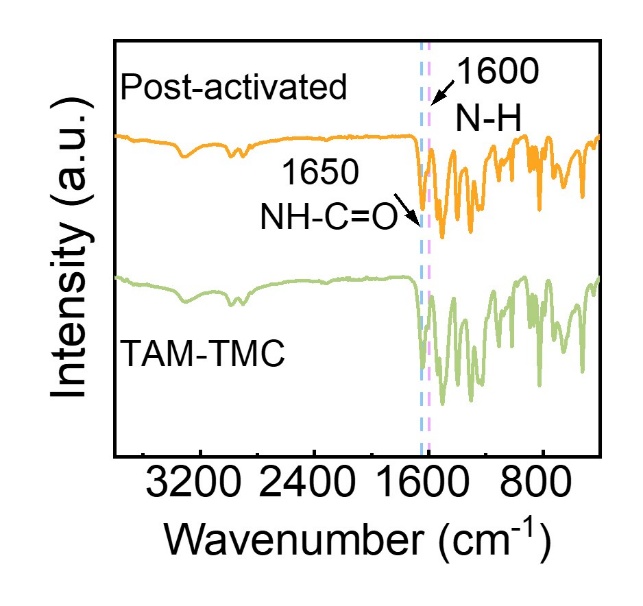


**Figure S24.** FT-IR spectrums of TAM-TMC membranes before and after activation.

**Table S1.** Different element contents of the membranes by XPS.


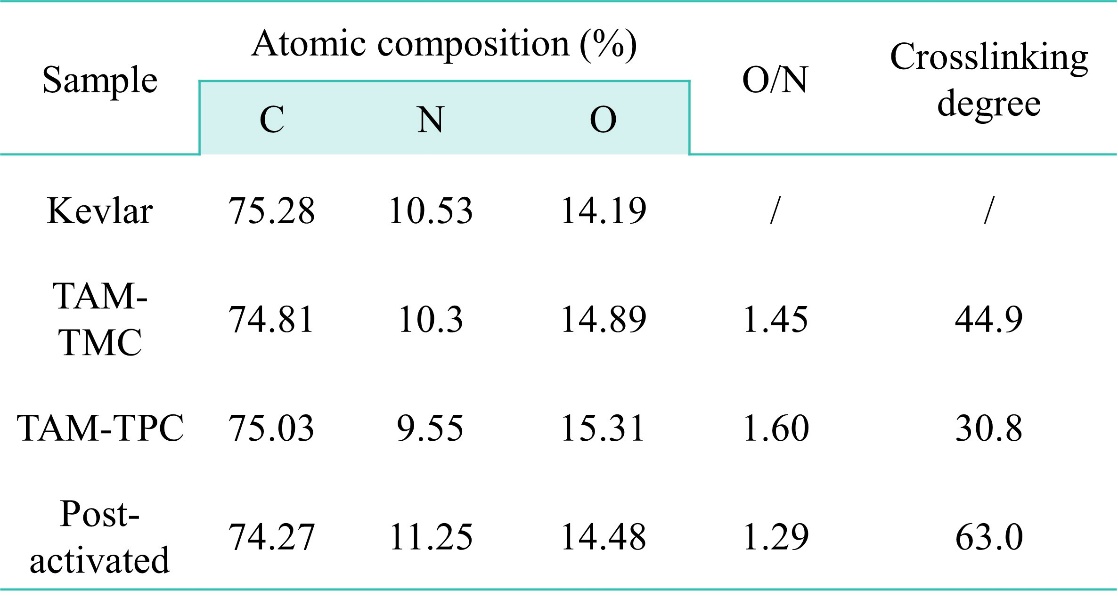


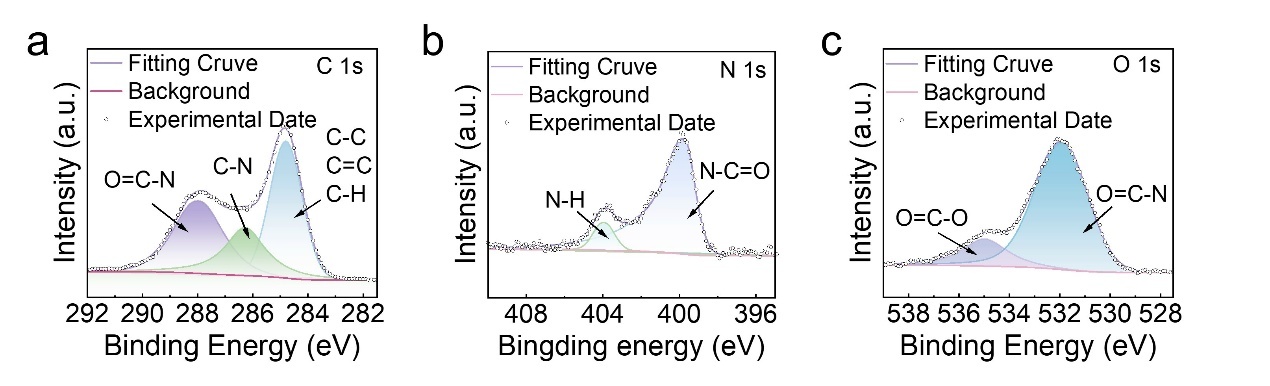


**Figure S25.** XPS data analysis of Kevlar hydrogels. (a) C1s spectrum; (b) N1s spectrum; (c) O1s spectrum.


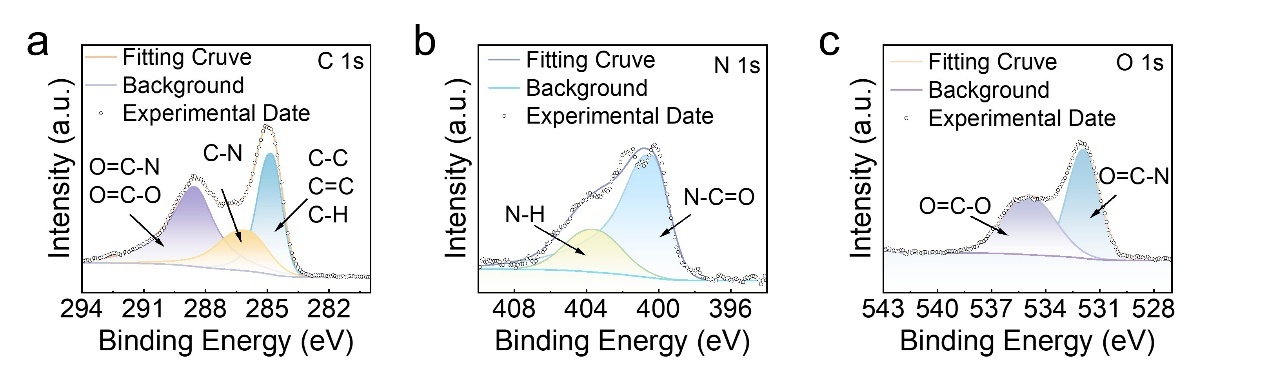


**Figure S26.** XPS data analysis of TAM-TMC membrane. (a) C1s spectrum; (b) N1s spectrum; (c) O1s spectrum.


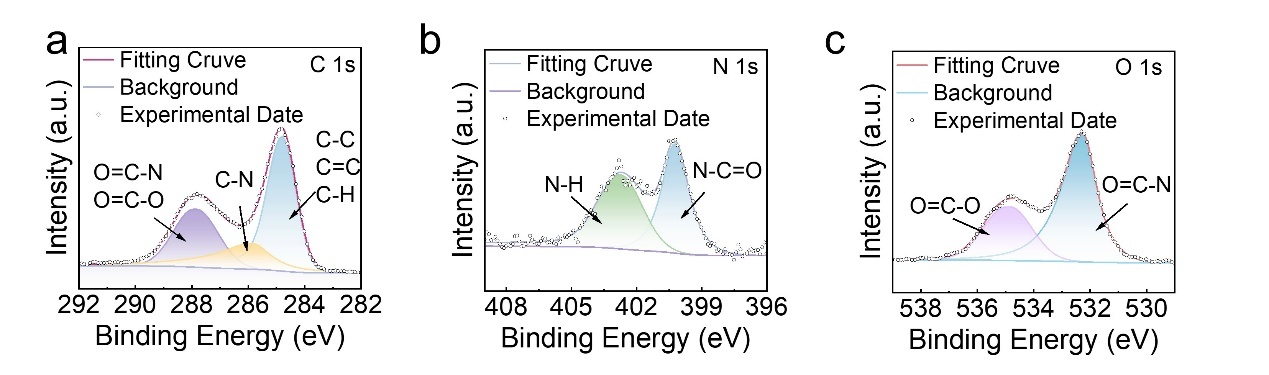


**Figure S27.** XPS data analysis of TAM-TPC membrane. (a) C1s spectrum; (b) N1s spectrum; (c) O1s spectrum.


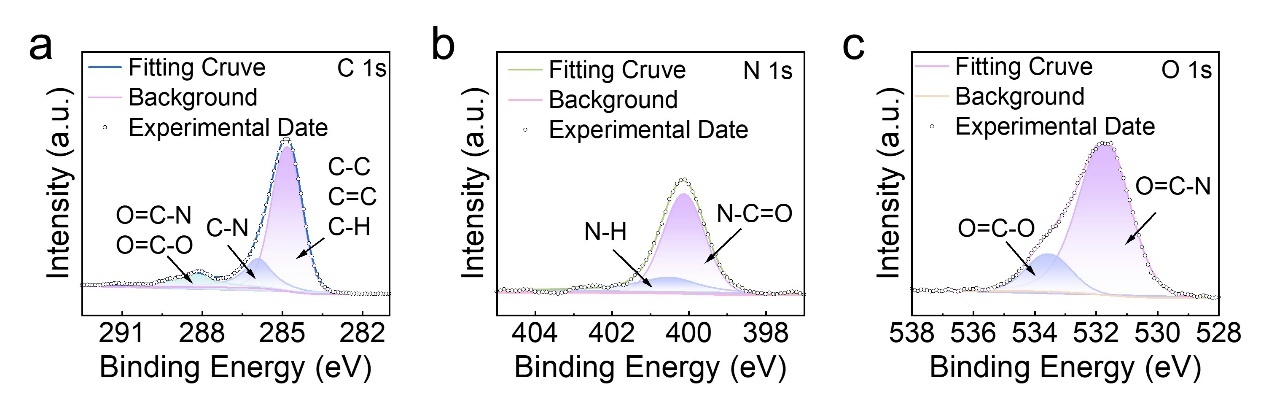


**Figure S28.** XPS data analysis of TAM-TMC membrane with DMF treatment 1h. (a) C1s spectrum; (b) N1s spectrum; (c) O1s spectrum.


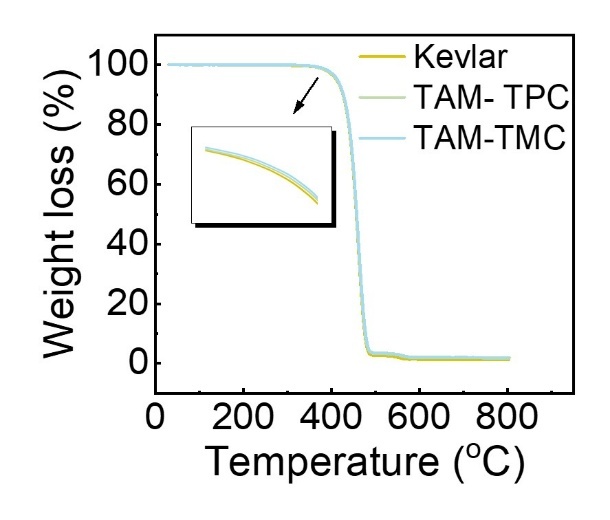


**Figure S29.** TGA curve analysis of PA and Kevlar membranes.


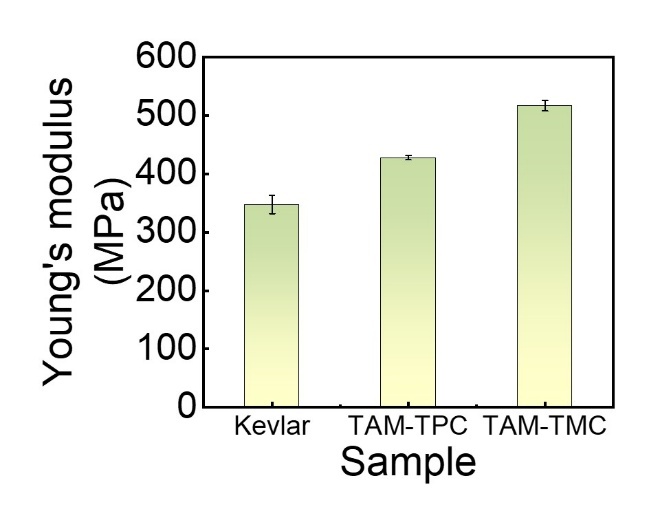


**Figure S30.** Young’s modulus histogram of prepared PA and Kevlar membranes.


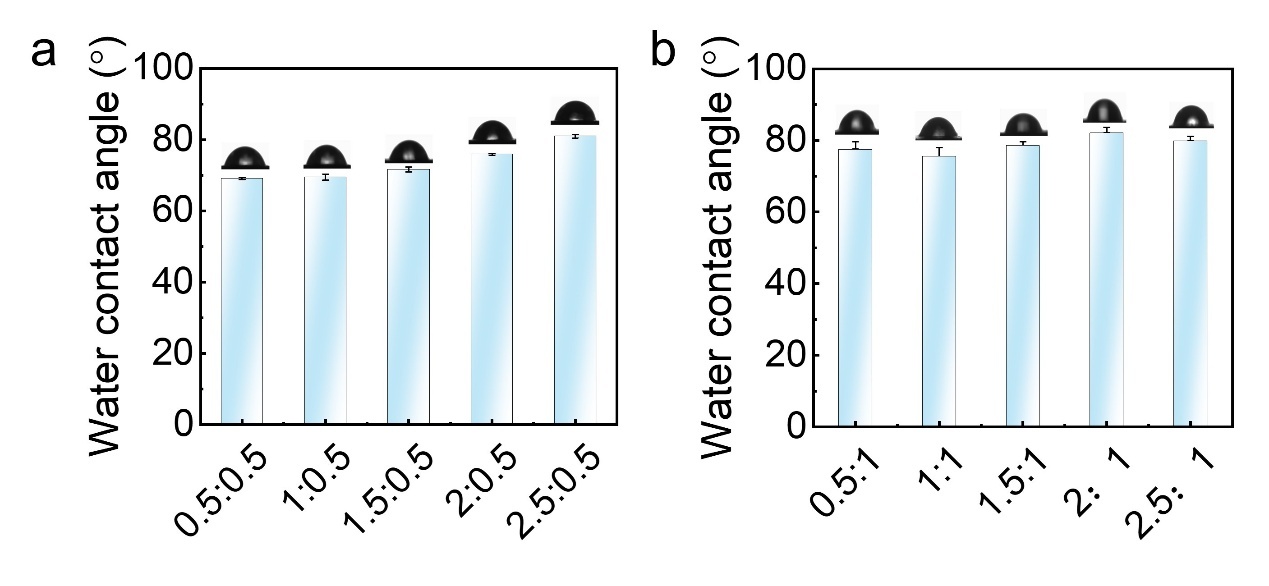


**Figure S31.** Water contact angle of TAM-TMC membranes. Measurement of water contact angle over different conditions.


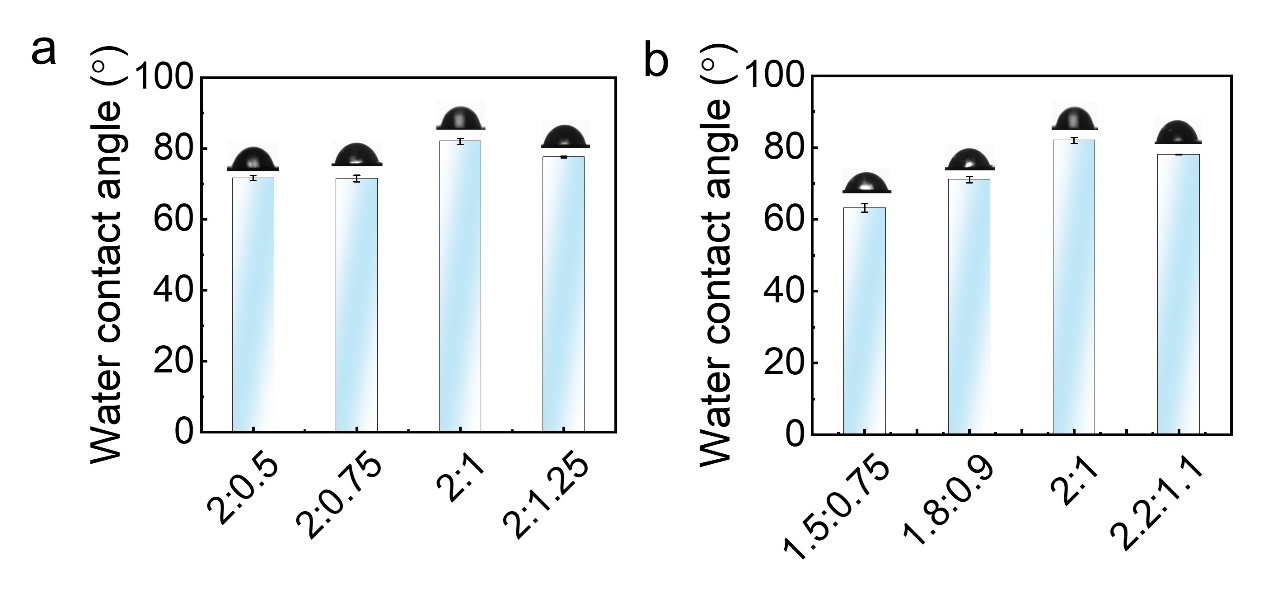


**Figure S32.** Water contact angle of TAM-TMC membranes. Measurement of water contact angle over different conditions.


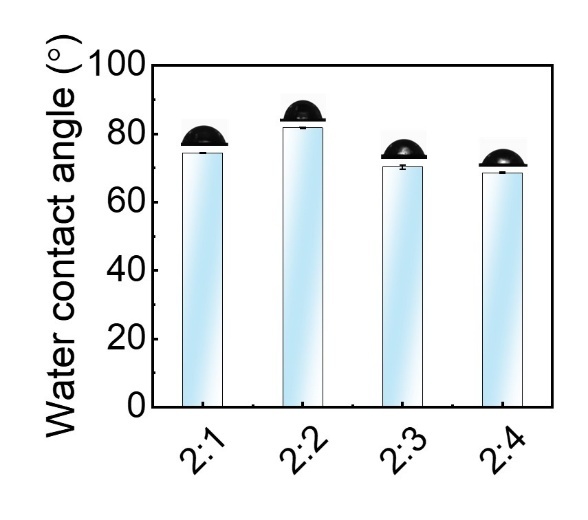


**Figure S33.** Water contact angle of TAM-TPC membranes. Measurement of water contact angle over different conditions.


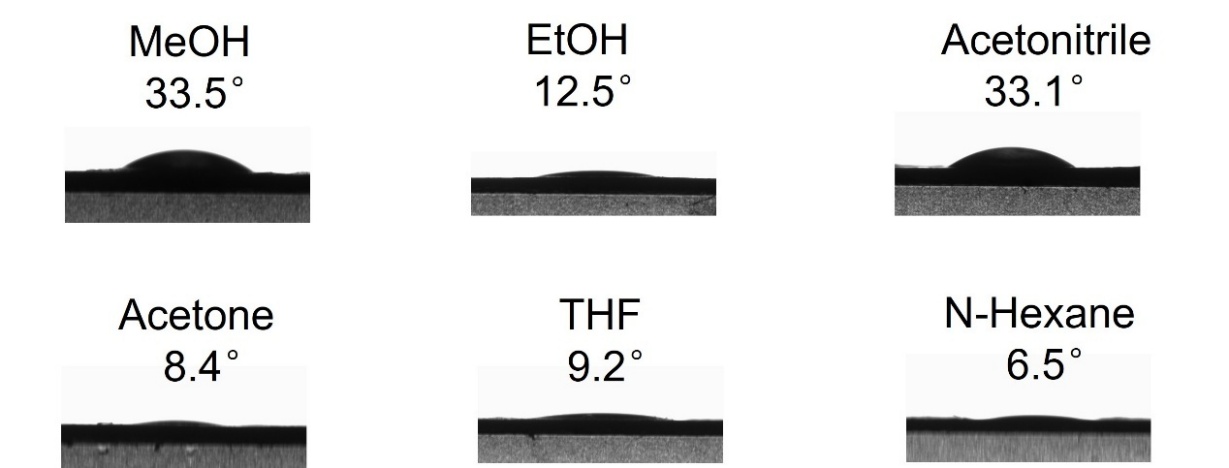


**Figure S34.** The contact angle images of TAM-TMC membrane in different polarity solvents.


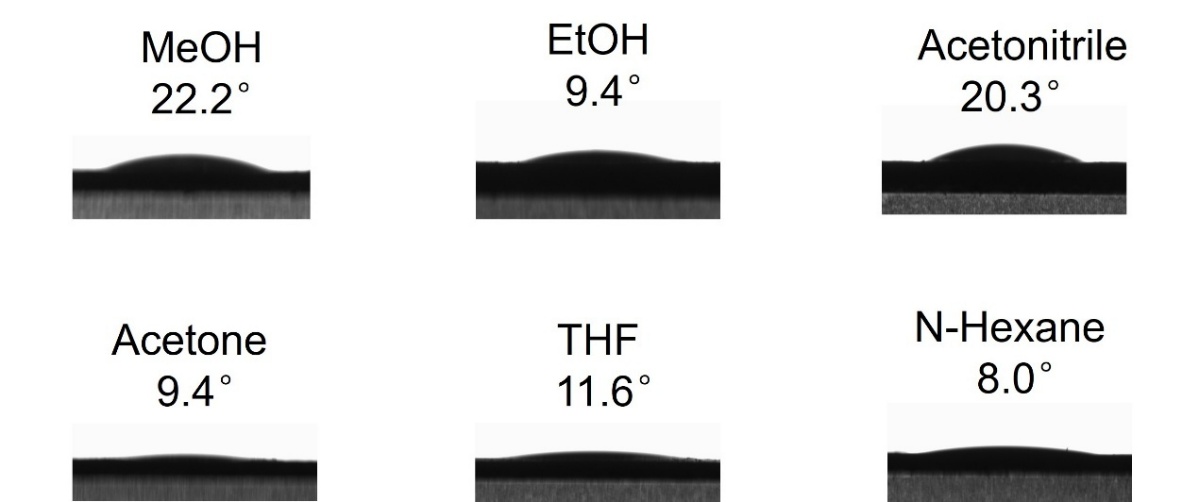


**Figure S35.** The contact angle images of TAM-TPC membrane in different polarity solvents.

**Table S2.** Detailed parameters of the solvents used in this study.^[9]^

δ= solubility parameter due to dipole forces.


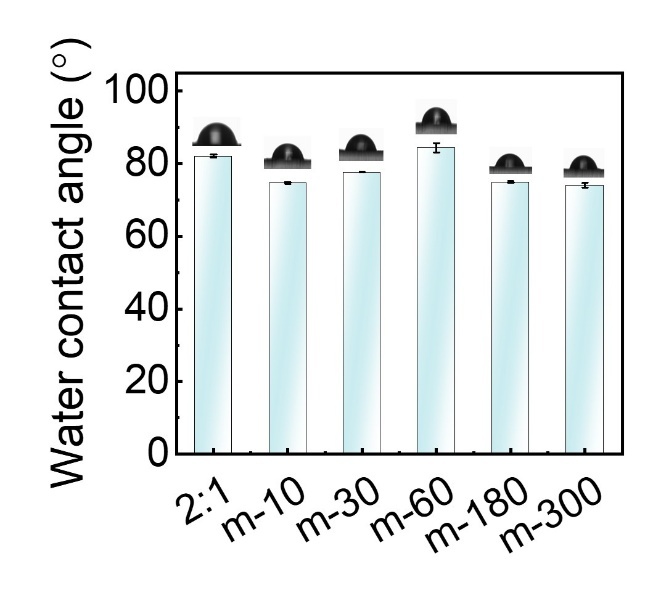


**Figure S36.** Water contact angle of TAM-TMC membranes with different DMF treatment time. Measurement of water contact angle over different conditions.


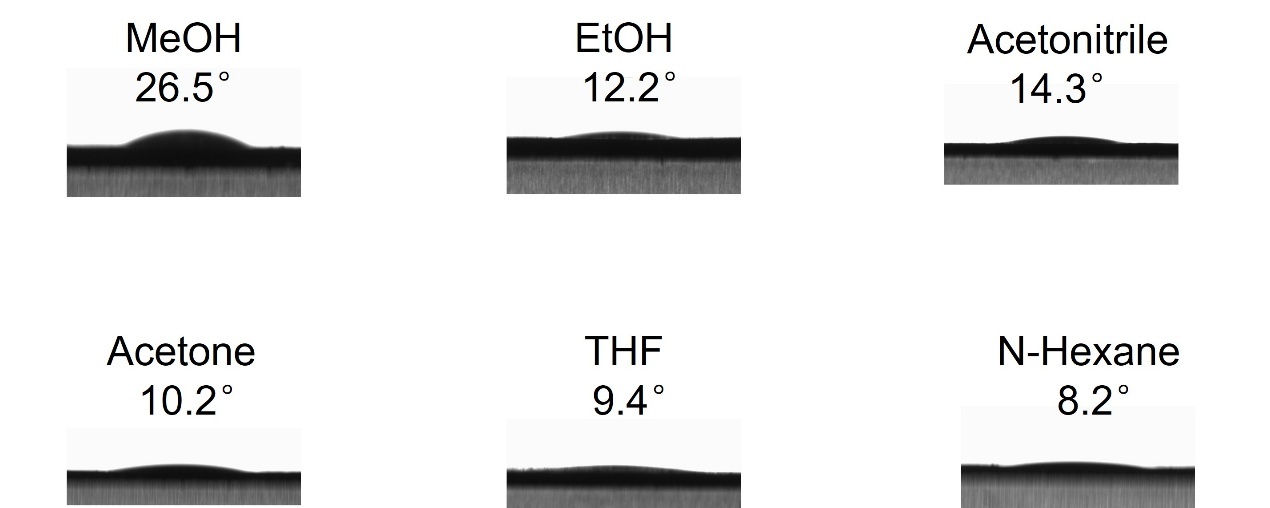


**Figure S37.** The contact angle images of TAM-TMC membrane with DMF treatment 1h in different polarity solvents.


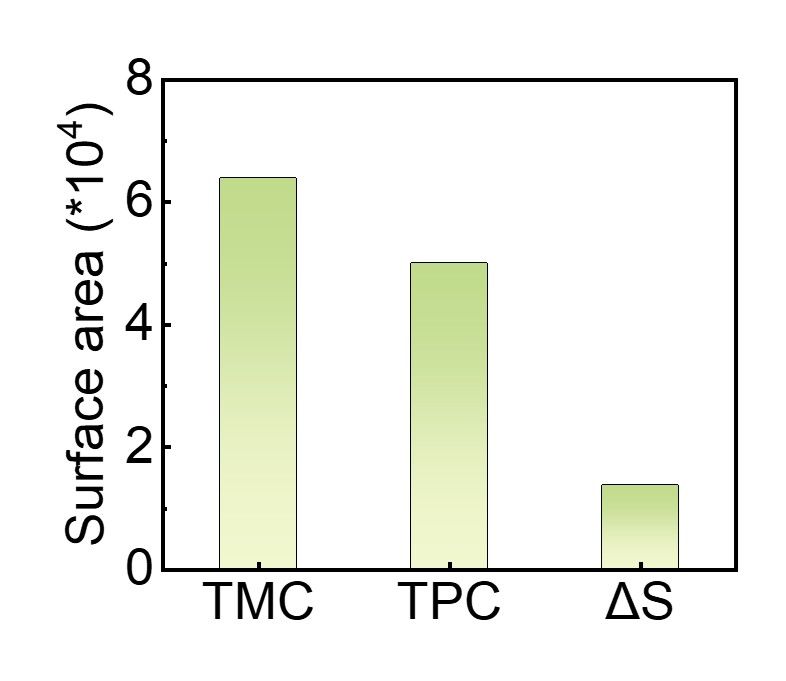


**Figure S38.** The internal surface area changes from TMC to TPC in polyamides.

**Table S3.** Simulated structural properties of TAM-TMC and TAM-TPC membranes.


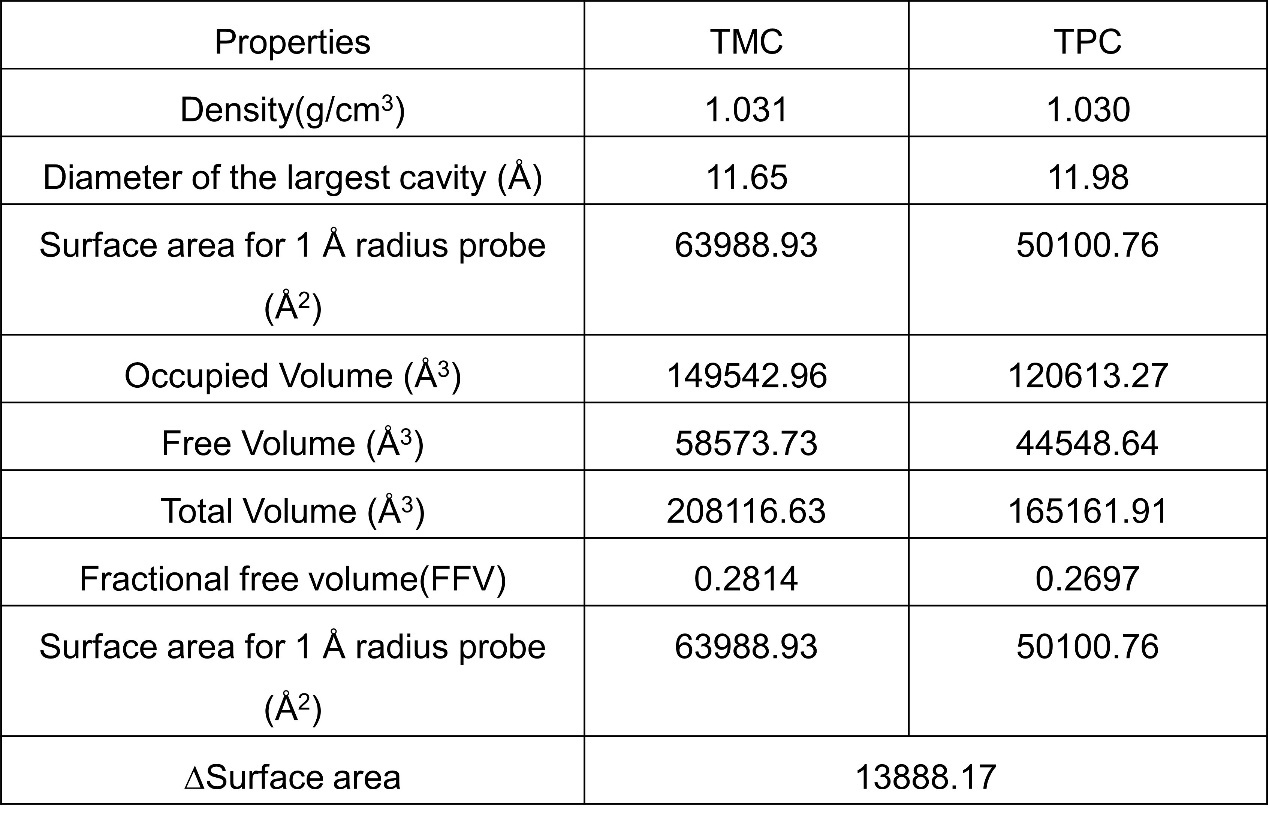


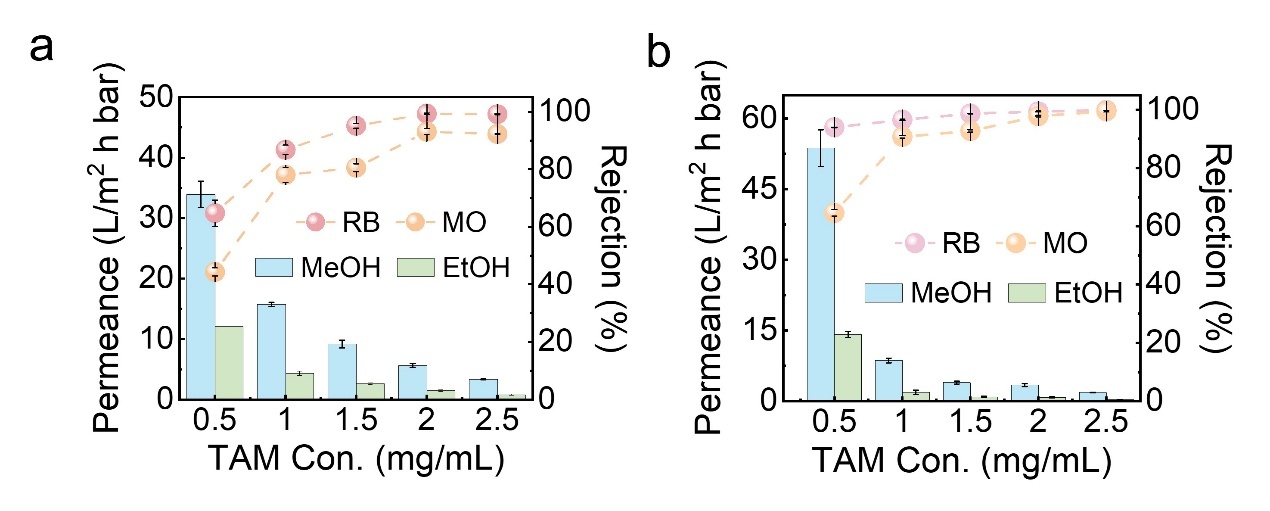


**Figure S39.** Modulation of TAM monomer concentrations to optimize the permeability and separability of TAM-TMC membrane at a fixed TMC concentration of (a) 0.05 w/v%; (b) 0.1 w/v%.


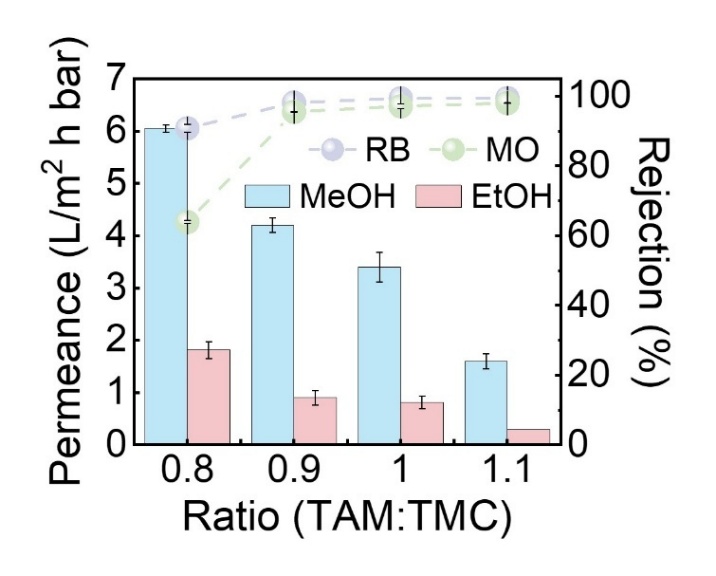


**Figure S40.** Regulation of the optimal ratio of TAM to TMC concentration to optimize the permeability and separability of TAM-TMC membrane.


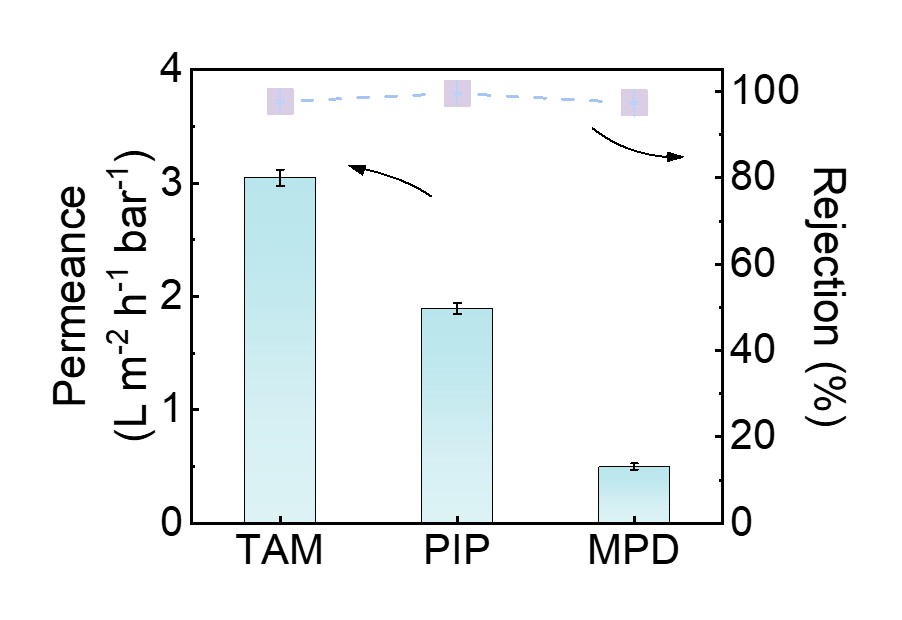


**Figure S41.** The performance of PA membranes prepared from different amine monomers under identical parameters.


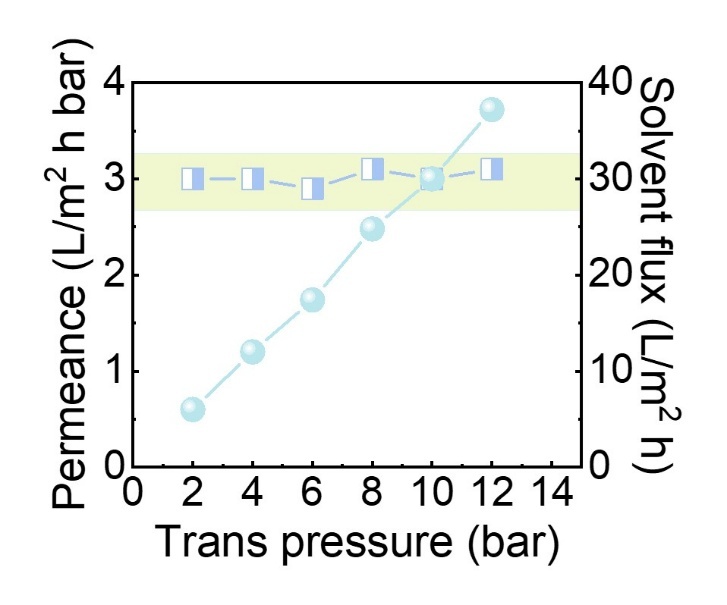


**Figure S42.** Methanol flux and permeance versus applied pressures for TAM-TMC composite membrane.


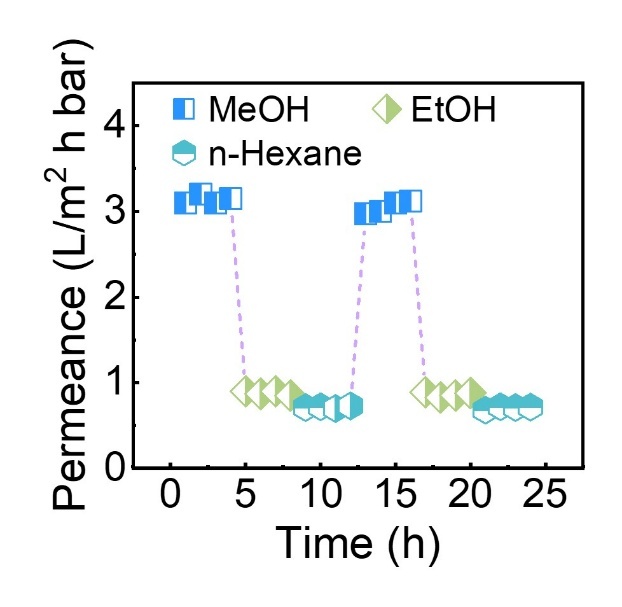


**Figure S43.** Stability of TAM-TMC membrane during cyclic test with solvents of different polarities.

**Table S4.** Structures of the small molecules used for filtration tests.^[10]^


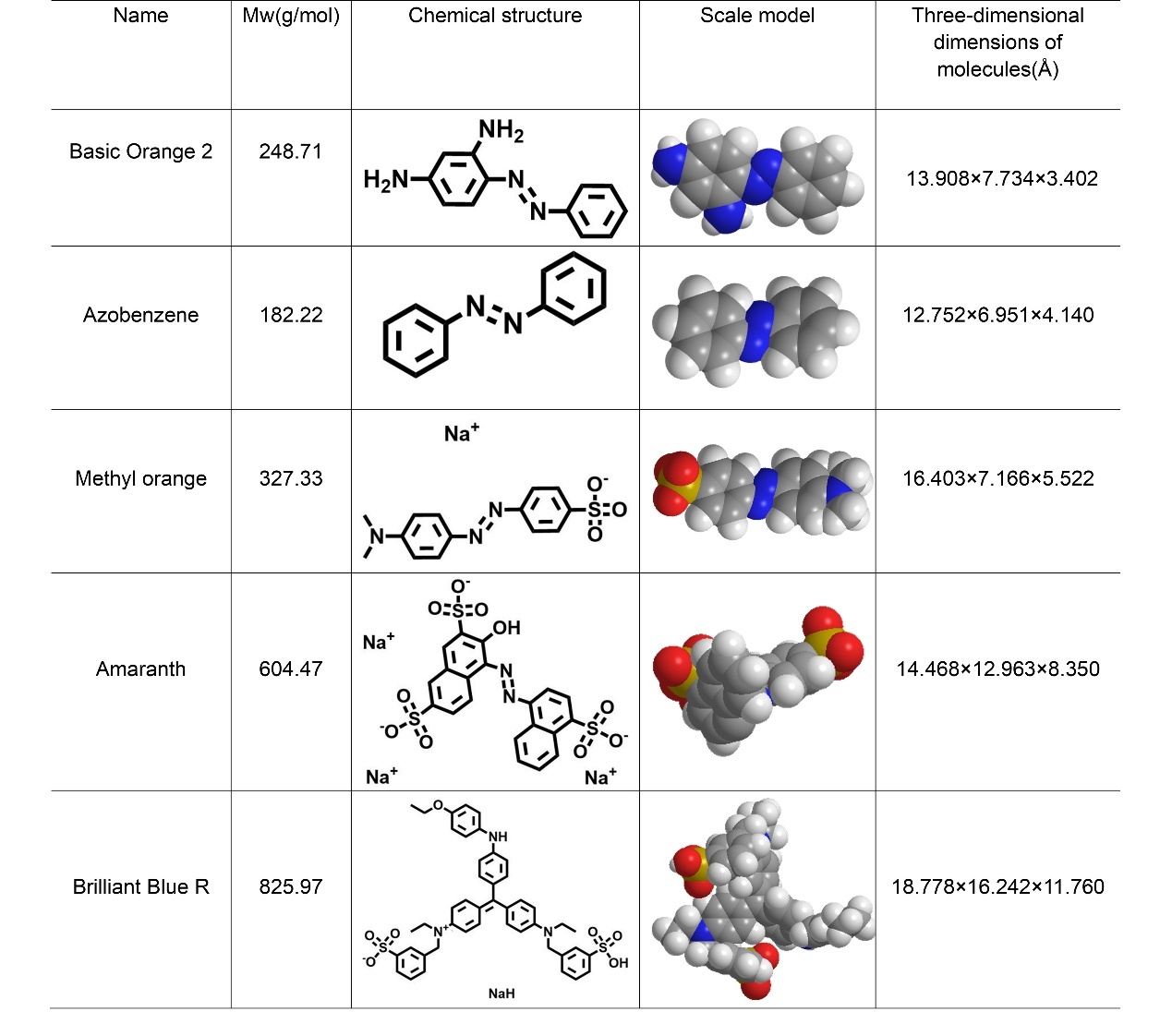


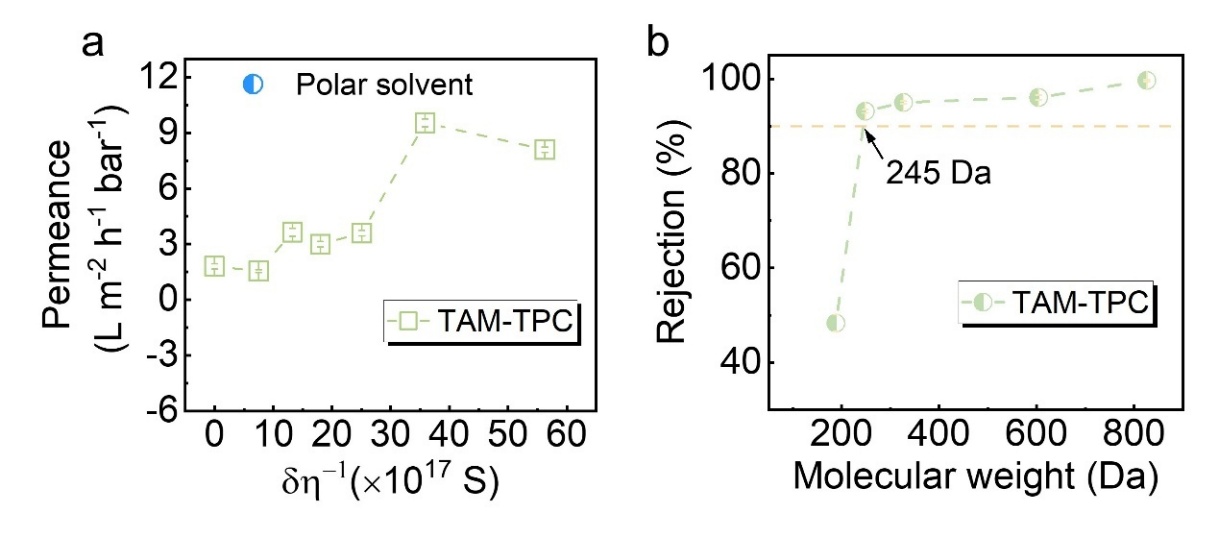


**Figure S44.** (a) Relationship of the permeance of different solvents with the solvent viscosity for TAM-TPC membranes. (b) Molecular rejection profile of the TAM-TPC membrane.


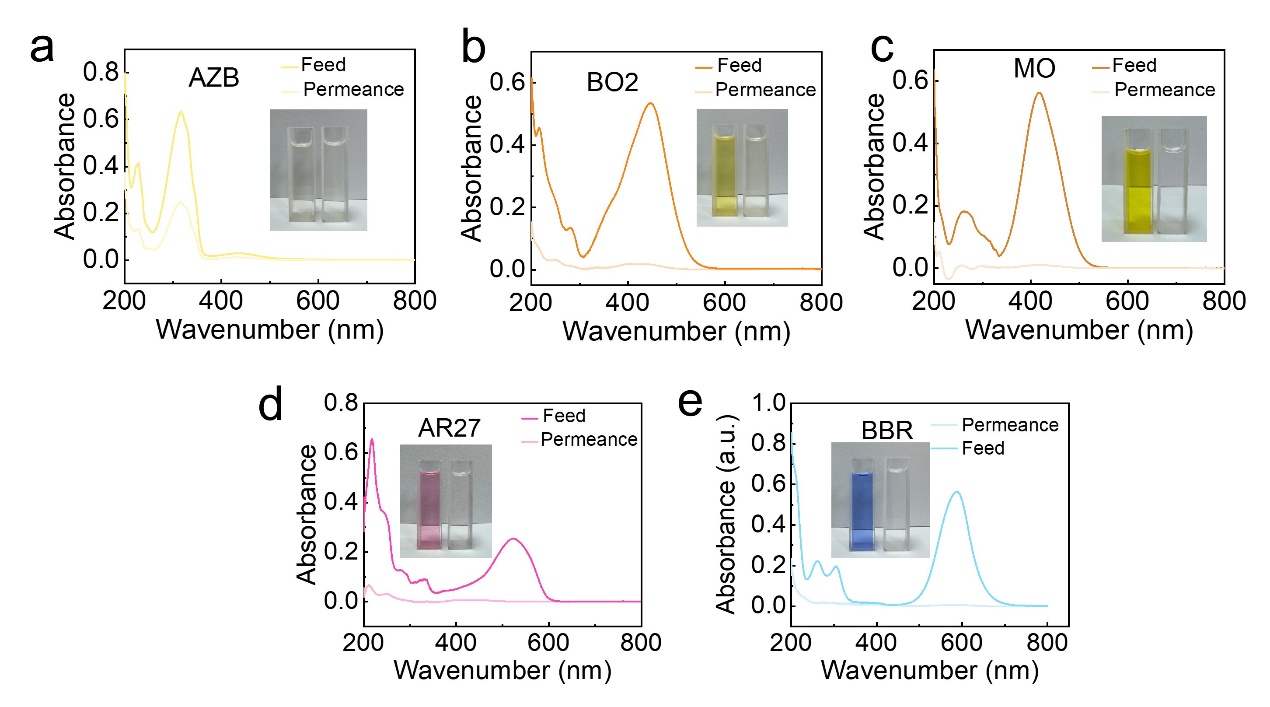


**Figure S45.** UV-vis spectra of the feeds and filtrates during the separation of small molecules by the TAM-TMC membrane: (a) AZB, (b) BO2, (c) MO, (d) AR27, (e) BBR.


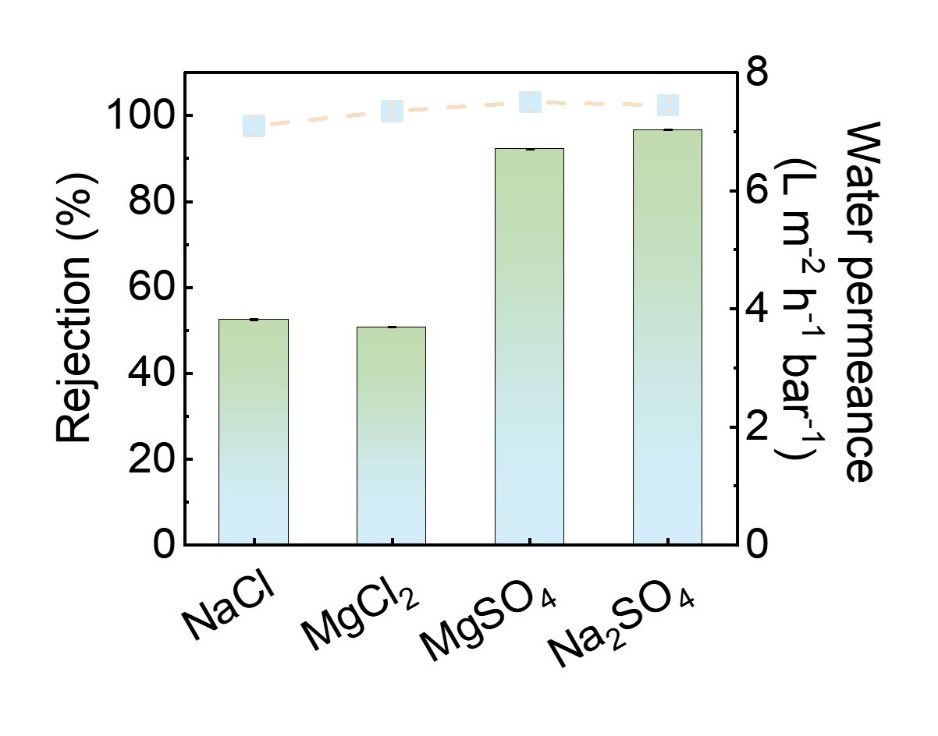


**Figure S46.** NF performance of activated TAM-TMC membranes for different inorganic salts.


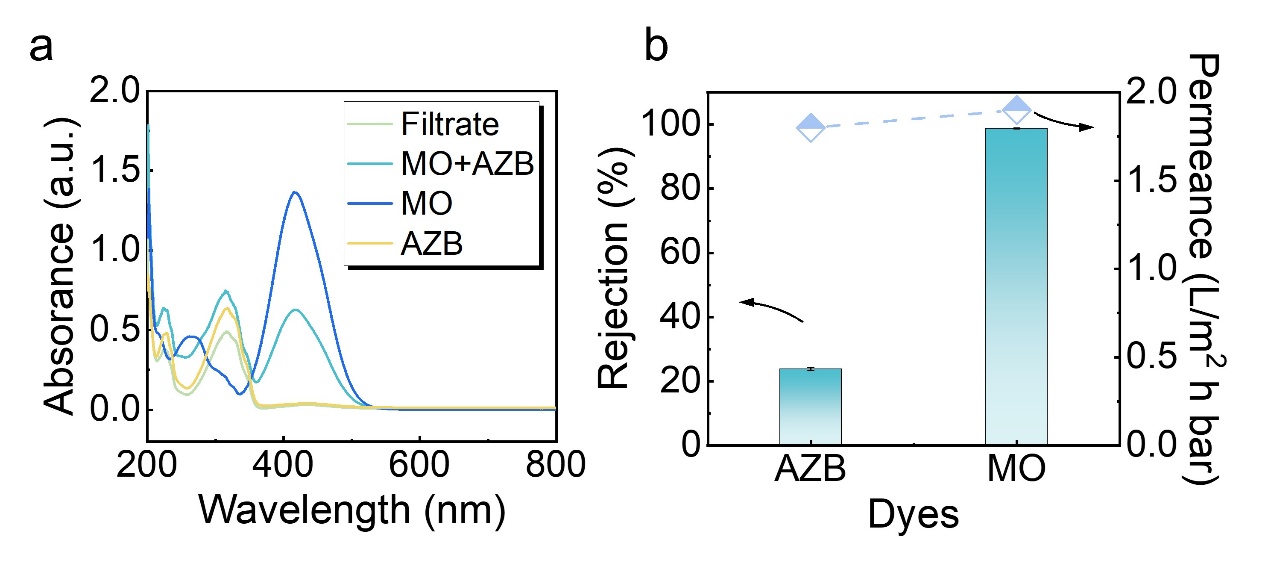


**Figure S47.** UV-vis spectra for the sieving of binary mixtures: (a) MO and AZB; (b) The separation performance of TAM-TMC membranes with DMF treatment 1h.

**Table S5.** Molecular structures of the pharmaceuticals used in this study.^[11]^


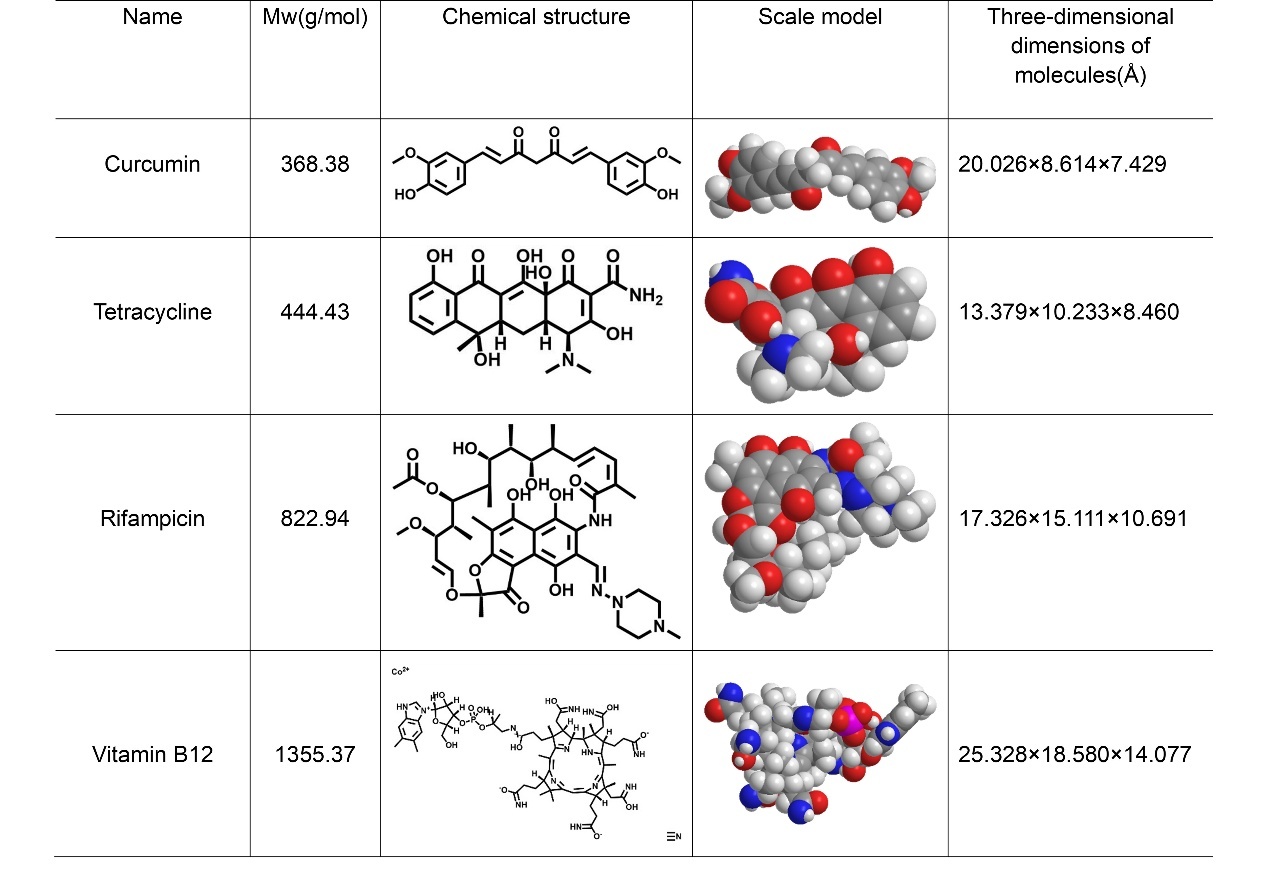


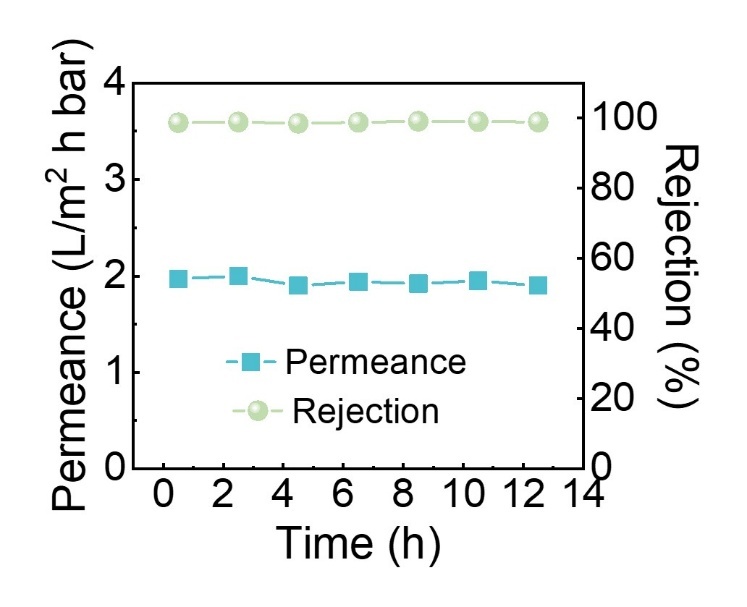


**Figure S48.** Long-term separation of vitamin B12 by the TAM-TMC membrane with DMF treatment 1h.


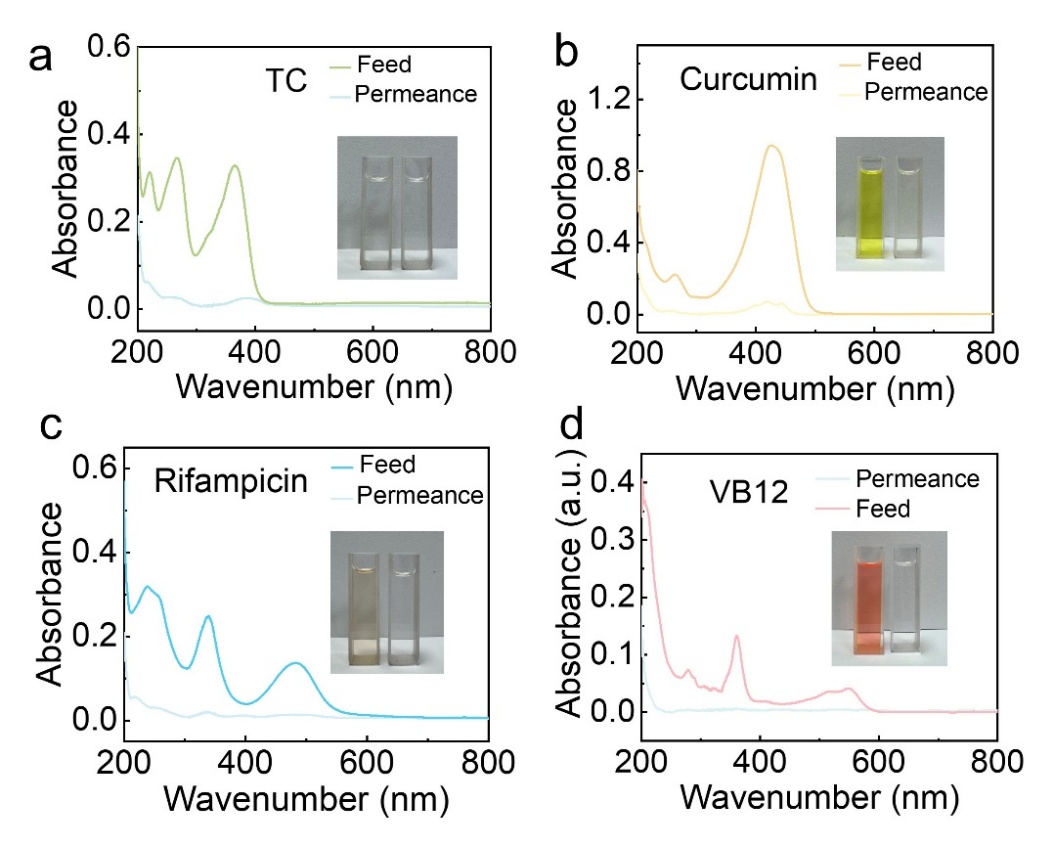


**Figure S49.** UV absorption spectra of drugs in ethanol before and after selectivity tests performed with TAM-TMC membranes with DMF treatment 1h.


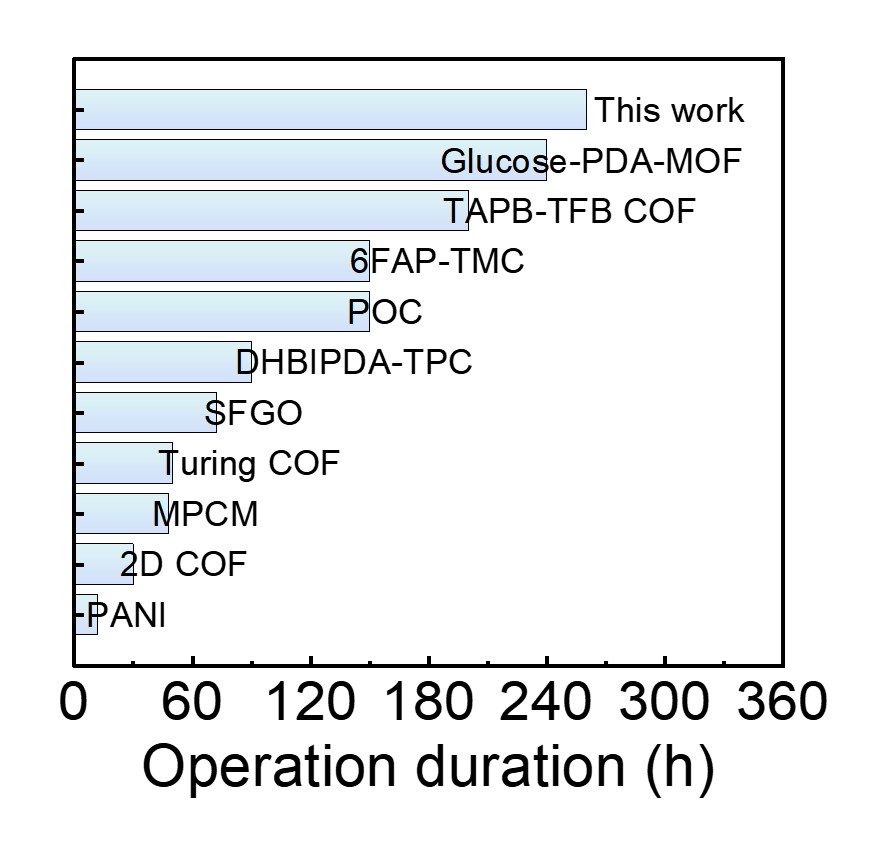


**Figure S50.** Comparison on the operation duration of our membrane and others.^[11b, 12]^


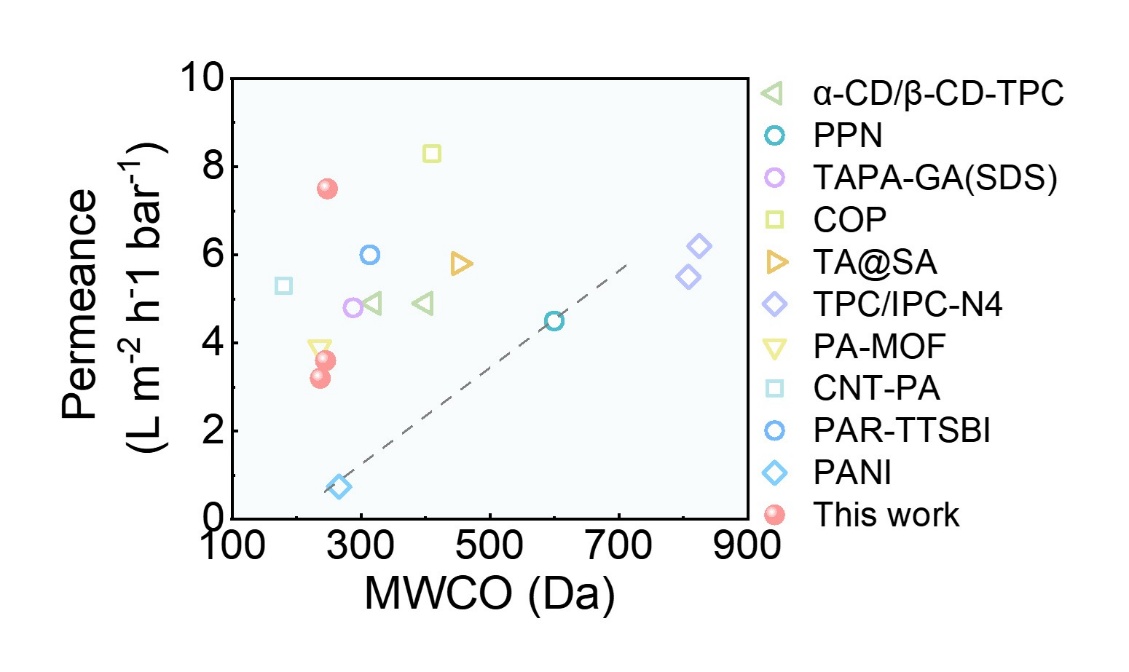


**Figure S51.** Separation performance of prepared 3D CON membranes in comparison with the recently reported nanofiltration membranes.

**Table S6.** Literature review. MeOH permeance and MWCO of polymeric membranes reported in the literature and prepared in this study.

| Membrane | MWCO | MeOH  Permeance  (LMH/bar) | Filtration type | Ref |
| --- | --- | --- | --- | --- |
| α-CD/TPC | 320 | 4.9 | dead-end | ^[13]^ |
| β-CD/TPC | 400 | 4.9 |  |  |
| TAPA-GA(SDS) | 288 | 4.8 | dead-end | ^[14]^ |
| TA@SA | 452.4 | 5.8 | dead-end | ^[15]^ |
| PPN | 600 | 4.5 | dead-end | ^[16]^ |
| COP | 410 | 8.3 | dead-end | ^[17]^ |
| PA-MOF | 236 | 3.9 | dead-end | ^[18]^ |
| TPC-N4 | 825 | 6.2 | dead-end | ^[19]^ |
| IPC-N4 | 808 | 5.5 |  |  |
| PAR-BINOL | 314 | 6 | dead-end | ^[20]^ |
| PANI | 266 | 0.74 | dead-end | ^[21]^ |
| CNT-PA | 180 | 5.3 | dead-end | ^[22]^ |
| TAM-TMC | 237 | 3.2 | dead-end | This work |
| TAM-TPC | 245 | 3.6 |  |  |
| Activated-TAM-TMC | 248 | 7.5 |  |  |

**Supplementary References**

[1] a) L. Ge, T. Chen, Z. Liu, Y. Li, H. Su, J. Zhu, Y. Zhang, B. V. der Bruggen, *Journal of Membrane Science* **2025**, 713, 123354; b) L. Ge, J. Li, G. Du, K. Sheng, J. Hou, B. Zhou, Y. Zhang, B. Van der Bruggen, J. Zhu, *Advanced Functional Materials* **2025**, n/a, 2504997.

[2] a) Z. Hao, X. Tian, V. Mankol, Q. Li, J. Wang, Z. Wang, S. Zhao, *Journal of Membrane Science* **2023**, 672, 121449; b) C. Liu, J. Yang, B.-B. Guo, S. Agarwal, A. Greiner, Z.-K. Xu, *Angewandte Chemie International Edition* **2021**, 60, 14636.

[3] a) S. Yu, Z. Liu, N. Xu, J. Chen, Y. Gao, *Analytical Sciences* **2020**, 36, 947; b) W. Yao, Y. Weng, J. M. Catchmark, *Cellulose* **2020**, 27, 5563.

[4] Y. Li, E. Wong, Z. Mai, B. Van der Bruggen, *Journal of Membrane Science* **2019**, 592, 117396.

[5] S. Han, Z. Lu, J. Zhu, Z. Mai, H. Matsuyama, T. He, Y. Zhang, *Nano Letters* **2024**, 24, 12382.

[6] D. Van Der Spoel, E. Lindahl, B. Hess, G. Groenhof, A. E. Mark, H. J. C. Berendsen, *Journal of Computational Chemistry* **2005**, 26, 1701.

[7] B. Hess, H. Bekker, H. J. C. Berendsen, J. G. E. M. Fraaije, *Journal of Computational Chemistry* **1997**, 18, 1463.

[8] N. Schmid, A. P. Eichenberger, A. Choutko, S. Riniker, M. Winger, A. E. Mark, W. F. van Gunsteren, *European Biophysics Journal* **2011**, 40, 843.

[9] a) X. Shi, H. Li, T. Chen, J. Ren, W. Zhao, B. C. Patra, C. Kang, Z. Zhang, D. Zhao, *Angewandte Chemie International Edition* **2025**, 64, e202421661; b) H. Yang, H. Zhang, C. Kang, C. Ji, D. Shi, D. Zhao, *Science Advances* 10, eads0260.

[10] a) Z. Jiang, R. Dong, A. M. Evans, N. Biere, M. A. Ebrahim, S. Li, D. Anselmetti, W. R. Dichtel, A. G. Livingston, *Nature* **2022**, 609, 58; b) H. Guo, F. Li, X. Shui, J. Wang, C. Fang, L. Zhu, *ACS Applied Materials & Interfaces* **2023**, 15, 37077.

[11] a) A. Yao, J. Hou, P. Dou, J. Du, Q. Sun, Z. Song, L. Liu, J. Guan, J. Liu, *Science Advances* 10, eado7687; b) C.-G. Jin, W.-H. Zhang, N. Tian, B. Wu, M.-J. Yin, Q.-F. An, *Angewandte Chemie International Edition* **2024**, 63, e202405891.

[12] a) L. Nie, K. Goh, Y. Wang, J. Lee, Y. Huang, H. E. Karahan, K. Zhou, M. D. Guiver, T.-H. Bae, *Science Advances* 6, eaaz9184; b) L. Cao, C. Chen, S. An, T. Xu, X. Liu, Z. Li, I. C. Chen, J. Miao, G. Li, Y. Han, Z. Lai, *Journal of the American Chemical Society* **2024**, 146, 21989; c) P. He, S. Zhao, C. Mao, Y. Wang, G. Ma, Z. Wang, J. Wang, *Chemical Engineering Journal* **2021**, 420, 129338; d) Y. Chen, W. Shi, S.-L. Li, M. Wang, J. Wang, S. Hao, G. Gong, C. Ye, N. B. McKeown, Y. Hu, *Advanced Functional Materials* **2024**, n/a, 2406430; e) F. Yang, J. Guo, C. Han, J. Huang, Z. Zhou, S.-P. Sun, Y. Zhang, L. Shao, *Science Advances* 10, eadr9260; f) Z. Song, L. Liu, Q. Sun, J. Du, J. Guan, P. Dou, R. Zhang, Z. Jiang, J. Liu, *Angewandte Chemie International Edition* **2024**, 63, e202409296; g) K. Xu, Y. Zheng, J. Zhou, Y. Zhao, X. Pang, L. Cheng, H. Wang, X. Zhang, R. Zhang, Z. Jiang, *Advanced Functional Materials* **2024**, n/a, 2417383; h) T. Huang, B. A. Moosa, P. Hoang, J. Liu, S. Chisca, G. Zhang, M. AlYami, N. M. Khashab, S. P. Nunes, *Nature Communications* **2020**, 11, 5882; i) Y. Zhang, X. Cheng, X. Jiang, J. J. Urban, C. H. Lau, S. Liu, L. Shao, *Materials Today* **2020**, 36, 40.

[13] J. Liu, D. Hua, Y. Zhang, S. Japip, T.-S. Chung, *Advanced Materials* **2018**, 30, 1705933.

[14] J. Du, Q. Sun, W. He, L. Liu, Z. Song, A. Yao, J. Ma, D. Cao, S. U. Hassan, J. Guan, J. Liu, *Advanced Materials* **2023**, 35, 2300975.

[15] F. Fang, P. Liu, W. Lin, L. O. Alimi, B. Moosa, E. Maltseva, N. M. Khashab, *Angewandte Chemie International Edition* **2024**, n/a, e202416050.

[16] C. Wang, C. Li, E. R. C. Rutledge, S. Che, J. Lee, A. J. Kalin, C. Zhang, H.-C. Zhou, Z.-H. Guo, L. Fang, *Journal of Materials Chemistry A* **2020**, 8, 15891.

[17] H. Guo, C. Fang, F. Li, W. Cui, R. Xiong, X. Yang, L. Zhu, *Materials Horizons* **2023**, 10, 5133.

[18] S. Sorribas, P. Gorgojo, C. Téllez, J. Coronas, A. G. Livingston, *Journal of the American Chemical Society* **2013**, 135, 15201.

[19] A. Yao, J. Du, Q. Sun, L. Liu, Z. Song, W. He, J. Liu, *ACS Nano* **2023**, 17, 22916.

[20] W. Fu, W. Zhang, H. Chen, S.-L. Li, W. Shi, Y. Hu, *Journal of Materials Chemistry A* **2021**, 9, 7180.

[21] A. Sarihan, S. Shahid, J. Shen, I. Amura, D. A. Patterson, E. A. C. Emanuelsson, *Journal of Membrane Science* **2019**, 579, 11.

[22] L. Deng, R. R. Gonzales, W. Fu, G. Xu, Q. Song, R. Takagi, H. Matsuyama, *Carbon* **2024**, 216, 118582.
